# Characterisation of *Thinopyrum bessarabicum* chromosomes through genome-wide introgressions into wheat

**DOI:** 10.1007/s00122-017-3009-y

**Published:** 2017-11-03

**Authors:** Surbhi Grewal, Caiyun Yang, Stella Hubbart Edwards, Duncan Scholefield, Stephen Ashling, Amanda J. Burridge, Ian P. King, Julie King

**Affiliations:** 1 Nottingham/BBSRC Wheat Research Centre, Division of Plant and Cop Sciences, School of Biosciences, The University of Nottingham, Sutton Bonington Campus, Loughborough, Leicestershire LE12 5RD UK; 2 Life Sciences, University of Bristol, Bristol, BS8 1TQ UK

## Abstract

**Key message:**

Genome-wide introgressions of *Thinopyrum bessarabicum* into wheat resulted in 12 recombinant lines. Cytological and molecular techniques allowed mapping of 1150 SNP markers across all seven chromosomes of the J genome.

**Abstract:**

*Thinopyrum bessarabicum* (2*n* = 2*x* = 14, JJ) is an important source for new genetic variation for wheat improvement due to its salinity tolerance and disease resistance. Its practical utilisation in wheat improvement can be facilitated through development of genome-wide introgressions leading to a variety of different wheat–*Th* *. bessarabicum* translocation lines. In this study, we report the generation of 12 such wheat–*Th* *. bessarabicum* recombinant lines, through two different crossing strategies, which were characterized using sequential single colour and multi-colour genomic in situ hybridization (sc-GISH and mc-GISH), multi-colour fluorescent in situ hybridization (mc-FISH) and single nucleotide polymorphic (SNP) DNA markers. We also detected 13 lines containing different *Th. bessarabicum* chromosome aberrations through sc-GISH. Through a combination of molecular and cytological analysis of all the 25 lines containing *Th. bessarabicum* recombinants and chromosome aberrations we were able to physically map 1150 SNP markers onto seven *Th. bessarabicum* J chromosomes which were divided into 36 segmental blocks. Comparative analysis of the physical map of *Th. bessarabicum* and the wheat genome showed that synteny between the two species is highly conserved at the macro-level and confirmed that *Th. bessarabicum* contains the 4/5 translocation also present in the A genome of wheat. These wheat–*Th* *. bessarabicum* recombinant lines and SNP markers provide a useful genetic resource for wheat improvement with the latter having a wider impact as a tool for detection of introgressions from other *Thinopyrum* species containing the J or a closely-related genome such as *Thinopyrum intermedium* (J^r^J^r^J^vs^J^vs^StSt) and *Thinopyrum elongatum* (E^e^E^e^), respectively.

**Electronic supplementary material:**

The online version of this article (10.1007/s00122-017-3009-y) contains supplementary material, which is available to authorized users.

## Introduction

Genetic diversity in wheat was reduced during domestication resulting in a narrow gene pool hindering the development of superior wheat varieties especially in the current climate of plateauing crop yields and dynamic biotic threats. However, some genetic variation can be introduced through introgressions from its distant wild relatives (Able and Langridge 2006; Feuillet et al. 2008; Gill et al. 2011; Zhang et al. 2017). These wild relatives are a largely unexploited source of agronomically important traits (Danilova et al. 2017; Friebe et al. 1996; Jauhar and Chibbar 1999; Schneider et al. 2008).

*Thinopyrum bessarabicum* (Savul. and Rayss) A. Lӧve (2*n* = 2*x* = 14, JJ) is a perennial maritime wheatgrass reported to possess salt tolerance (King et al. 1997a) and disease resistance (Xu et al. 2009), making it a valuable source of genetic variation for wheat improvement. High-salinity tolerance was found in hexaploid amphiploids (2*n* = 6*x* = 42, AABBJJ) derived from Chinese Spring and *Th. bessarabicum* (Hassani et al. 2010; King et al. 1997b). Wheat–*Th* *. bessarabicum* chromosome addition lines have also been developed and studied allowing gene discovery for useful traits that can be further utilised in wheat improvement programmes (King et al. 1996; William and Mujeeb-Kazi 1993, 1995; Xu et al. 2009; Zhang et al. 2002) but such addition and substitution lines are generally unsuitable for agronomic purposes, as many unwanted genes are introduced along with those giving rise to the trait of interest.

Homoeologous recombination can reduce the size of the wild relative segment including the gene(s) of interest incorporated into the wheat chromosome. However, presence of the *Ph1* locus makes it difficult to induce homoeologous recombination between chromosomes of wheat and its wild relatives, and therefore, there has been limited utilization of alien genes in wheat (Griffiths et al. 2006; Riley and Chapman 1958). Several approaches have been used to allow introgression of wild relative chromatin into wheat, including *ph1* mutant (King et al. 2017; Zhao et al. 2013), irradiation (Bie et al. 2007; Chen et al. 2013), tissue culture (Banks et al. 1995; Lapitan et al. 1984; Larkin and Scowcroft 1981), spontaneous translocations (Liu et al. 2013) and gametocidal genes (Endo 2007; Masoudi-Nejad et al. 2002). A number of these approaches have also been used to develop wheat–*Th*. *bessarabicum* translocation and recombinant lines (Ardalani et al. 2016; Ghazali et al. 2015; King et al. 1993a; Qi et al. 2010; Zhuang et al. 2004). These translocations/recombinants have been further characterised using biochemical, cytological and molecular techniques (King et al. 1993b; Patokar et al. 2016; Pu et al. 2015; Shen et al. 2013; William and Mujeeb-Kazi 1993).

The major problem in developing wheat-wild relative introgressions is the selection of recombinants, which occur only at low frequency. Such selection would be greatly enhanced by the availability of genetic markers spaced along the wild relative chromosome since conventional cytological methods are difficult to use to identify small segmental recombinants. In previous studies, molecular resources used to characterise wheat–*Th*. *bessarabicum* recombinants and translocations have included random amplified polymorphic DNA (RAPD) markers (King et al. 1993b), expressed sequence tag (EST)-derived sequences (Chen et al. 2007; Luan et al. 2010; Shen et al. 2013) and PCR based Landmark Unique Gene (PLUG) markers (Ardalani et al. 2016; Ghazali et al. 2015). However, these techniques as well as just cytological detection are low-throughput and have limited success. The development of next-generation sequencing technologies and high-throughput single nucleotide polymorphism (SNP) markers and corresponding SNP-arrays, has enabled faster, more accurate detection of introgressions from wild relatives into wheat (King et al. 2017; Tiwari et al. 2014, 2015). This high-throughput genotyping approach can then be complemented by genomic in situ hybridization (GISH), which allows the direct visualization of alien chromatin (Schwarzacher et al. 1992).

In this study, we have developed a resource of SNP markers spread across all seven *Th. bessarabicum* chromosomes which were used to identify 12 wheat–*Th*. *bessarabicum* recombinants and detect intact *Th. bessarabicum* chromosomes alongside spontaneous structural aberrations in introgression lines. These wheat–*Th*. *bessarabicum* introgression lines were developed using two different crossing strategies (King et al., 2017). A combination of cytological methods and molecular marker analysis of these recombinants and aberrations allowed physical characterisation of all *Th. bessarabicum* chromosomes into 36 segmental blocks of 1150 SNP markers. Development and physical localisation of such resources of high-density molecular markers specific for wild relative chromosomes will greatly improve the efficiency of wild relative introgressions into wheat.

## Materials and methods

### Plant materials

**Fig. 1:**
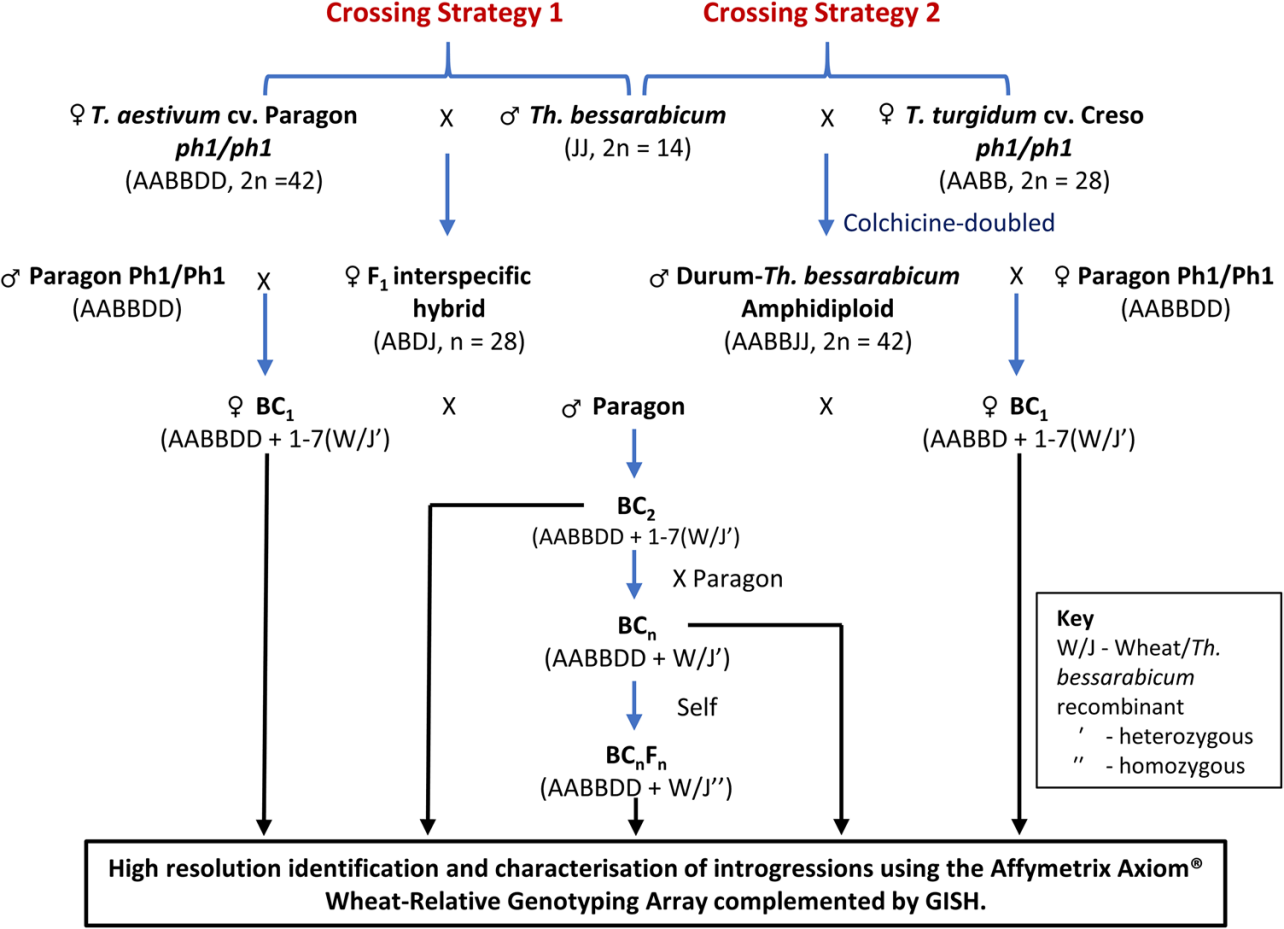
A crossing programme showing both strategies employed to generate wheat-*Th. bessarabicum* recombinants. All backcross populations were genotyped with the Affymetrix Axiom^®^ Wheat-Relative Genotyping Array to identify and characterise any introgressions from *Th. bessarabicum* into wheat which were also confirmed with GISH

To generate introgressions, two crossing strategies were employed as illustrated in Fig. 1. In the first (Crossing Strategy 1), hexaploid wheat *Triticum aestivum* cv. Paragon *ph1/ph1* mutant (2*n* = 6*x* = 42) was pollinated with *Th. bessarabicum* (accession PI 531712, obtained from United Stated Department of Agriculture, USDA; 2*n* = 2*x* = 14) to produce F_1_ interspecific hybrids. The second crossing program (Crossing Strategy 2) exploited colchicine-doubled hybrids between tetraploid wheat *Triticum turgidum* L. cv. Creso *ph1/ph1* mutant (2*n* = 4*x* = 28) and *Th. bessarabicum* (King et al. 1993a) obtained from the Germplasm Resource Unit (GRU) at the John Innes Centre. In this work, introgression of genetic variation from *Th. bessarabicum* into wheat was expected to occur when the chromosomes of the two species recombine in the absence of the *Ph1* pairing locus during gametogenesis in these interspecific F_1_ hybrids. This recombination would result in the production of gametes which carry *Th. bessarabicum*/wheat recombinant chromosomes (the subsequent transmission of these recombinant chromosomes to their progeny leads to the generation of *Th. bessarabicum*/wheat introgressions). Hybrids from both strategies were grown to maturity and backcrossed. F_1_s from the first strategy were used as the female and backcrossed with Paragon, carrying the wild-type *Ph1* locus intact, to generate a BC_1_ population. In the second strategy, the amphidiploids were used as the pollen donor and backcrossed onto hexaploid wheat Paragon *Ph1/Ph1* to produce BC_1_ plants. The BC_1_ individuals from both strategies and their resulting progenies were then recurrently pollinated with Paragon *Ph1/Ph1* to produce BC_2_, BC_3_ and BC_4_ populations. Three heads from each plant in each backcross population were bagged to allow self-fertilisation. Cross fertility was calculated as the number of crosses setting seed.

Thirteen wheat–*Th*. *bessarabicum* derivatives were used for allocating markers to their corresponding chromosomal locations in this study. Five of these derivatives were obtained from the GRU and included 4 disomic addition lines, DA1J-1 (WPGS id#28181), DA2J-1 (WPGS id#28183), DA4J-1 (WPGS id#28184), DA5J-1 (WPGS id#28185) and 1 ditelosomic addition line DtA5JS (WPGS id#28186). One translocation line, T2BS∙2BL-2JL [Line 1176; Patokar et al. (2016)], and 7 disomic addition lines DA1J-2 (GID: 1784842), DA2J-2 (GID: 184896), DA3J (GID: 1807809), DA4J-2 (GID: 1803509), DA5J-2 (GID: 1803460), DA6J (GID: 229186), DA7J (GID: 1803417) were kindly provided by Dr. Kishii Masahiro of the International Maize and Wheat Improvement Center (CIMMYT, Mexico). A further 3 wheat–*Th*. *bessarabicum* disomic addition lines WPGS id#28187, WPGS id#28188 and WPGS id#28189 were obtained from the GRU with unknown chromosome constitutions.

### Cytogenetic analysis

The protocol for genomic in situ hybridization (GISH) was as described in Zhang et al. (2013), Grewal et al. (2017) and in King et al. (2017). In summary, genomic DNAs from young leaves of the three putative diploid progenitors of bread wheat, i.e. *T. urartu* (A genome), *Ae. speltoides* (B genome), and *Ae. tauschii* (D genome) and of *Th. bessarabicum*, were isolated using extraction buffer [0.1 M Tris–HCl (pH 7.5), 0.05 M EDTA (pH 8.0), 1.25% SDS]. Samples were incubated at 65 °C for 1 h before being placed on ice and mixed with ice cold 6 M NH_4_C_2_H_3_O for 15 min. The samples were then spun down, the supernatant mixed with isopropanol to pellet the DNA and the isolated DNA further purified with phenol/chloroform. The genomic DNA of *Th. bessarabicum* and *T. urartu* were labelled by nick translation with Chroma Tide Alexa Fluor 488-5-dUTP (Invitrogen; C11397; green). Genomic DNA of *Ae. tauschii* was labelled with Alexa Fluor 594-5-dUTP (Invitrogen; C11400; red). Genomic DNAs of *Ae. speltoides* and *T. aestivum* cv. Paragon were fragmented to 300–500 bp in a heat block at 100 °C. Preparation of chromosome spreads was as described in Grewal et al. (2017) and in King et al. (2017).

Slides were initially probed, for single colour GISH (sc-GISH), using labelled genomic DNA of *Th. bessarabicum* (100 ng) and fragmented genomic DNA of Paragon (3000 ng) as blocker (1:30 per slide) to detect the *Th. bessarabicum* introgressions. The slides were bleached (dipped in 2 × SSC to remove the coverslip, transferred to 4 × SSC for 5 min and air dried in the light) and re-probed, for multi-colour GISH (mc-GISH) with labelled DNAs of *T. urartu* (100 ng) and *Ae. tauschii* (200 ng) and fragmented DNA of *Ae. speltoides* (3000 ng) as blocker (1:2:30 per slide) to detect the AABBDD genomes of wheat. All slides were counterstained with DAPI and analysed using a high-throughput, fully automated Zeiss Axio Imager.Z2 upright epifluorescence microscope (Carl Zeiss Ltd, Oberkochen, Germany) with filters for DAPI (blue), Alexa Fluor 488 (green) and Alexa Fluor 594 (red). Photographs were taken using a MetaSystems Coolcube 1 m CCD camera. Further slide analysis was carried out using Metafer (automated metaphase image capture) and ISIS (image processing) software (Metasystems GmbH, Altlussheim, Germany). This system enabled the fully automated capture of high and low power fluorescent images of root tip metaphase spreads. Where a sc-GISH image could not be obtained for the backcross line showing a *Th. bessarabicum* segment in the genotyping, its self-fertilised seed was used to obtain a sc-GISH image of the recombinant chromosome or structural aberration.

For multi-colour fluorescence in situ hybridization (mc-FISH), two repetitive DNA sequences pSc119.2 (McIntyre et al. 1990), and pAs.1 (Rayburn and Gill 1986) were labelled with Alexa Fluor 488-5-dUTP (green) and Alexa Fluor 594-5-dUTP (red), respectively, and hybridized to the slides prior to sc-GISH. Some slides were then bleached as described above and sequentially re-probed for sc-GISH and mc-GISH.

### Genotyping via an Axiom^®^ SNP array

The Nottingham/BBSRC Wheat Research Centre (WRC) is presently engaged in the genome-wide introgression of genetic variation from wild relatives into wheat. To detect introgressed chromosomes and chromosome segments from these wild relatives into wheat, an array of circa 35 K SNPs, known as the Axiom^®^ Wheat-Relative Genotyping Array, has been developed (King et al. 2017; Winfield et al. 2016). In summary, the array is composed of SNPs each showing polymorphism for the wild relatives relative to the wheat genotypes under study. All the SNPs incorporated in this array formed part of the Axiom^®^ 820 K SNP array (Winfield et al. 2016). The data-set for the Axiom^®^ 820 K array is available from http://www.cerealsdb.uk.net (Wilkinson et al. 2012, 2016). Table 2 shows the number of putative SNPs, for each linkage group, between *Th. bessarabicum* and the wheat genotypes included on the array. This 384-format genotyping array facilitates cost-effective, high-throughput, high resolution screening of introgressions that are being generated from wild relatives, including *Th. bessarabicum.*

The Axiom^®^ Wheat-Relative Genotyping Array was used to genotype 447 samples in total. Control samples included three replicates of each of the three parental lines, i.e. wheat cvs. Paragon and Creso and the wild relative *Th. bessarabicum*; one 2JL translocation line and 12 wheat–*Th*. *bessarabicum* disomic addition lines. Call rate for a sample was calculated as the percentage of the number of SNP probes on the array that resulted in a definitive genotype call (AA, AB, BB). The equipment, software, procedures and criteria used for this genotyping are as described by King et al. (2017).

### Physical mapping of *Th. bessarabicum* chromosomes

Individuals from a backcross population between wheat and *Th. bessarabicum* were genotyped with the Axiom^®^ Wheat-Relative Genotyping Array. Along with triplicates of all three parental lines from both crossing strategies, 422 lines comprising BC_1_, BC_2_, BC_3_ and BC_4_ populations of *Th. bessarabicum* and 16 wheat–*Th*. *bessarabicum* derivatives were genotyped altogether. As described in the methodology by King et al. (2017), only the Poly High Resolution (PHR) SNP markers, which were co-dominant and polymorphic and generated calls (that qualified as the minor allele) for at least 2 of the 3 replicates of *Th. bessarabicum*, were used for further marker analysis. SNP markers which showed (1) heterozygous calls for either parent(s), (2) no polymorphism between the wheat parents and *Th. bessarabicum* and/or (3) no calls for either parent(s) were removed using Flapjack™ (Milne et al. 2010; v.1.14.09.24). The resulting markers were sorted into linkage groups in JoinMap^®^ 4.0 (Van Ooijen 2011) with a LOD score of 40 using the genotype classification code ‘(a,h)’, where ‘a’ is the genotype of the first parent and ‘h’ is the genotype of the F_1_. ‘BCpxFy’ was used as the population code for each dataset which donates an advanced backcross inbred line family, where the backcross parent *p* had genotype ‘a’, *x* is the number of backcrosses including the one for creating the BC_1_ and *y* is the number of selfings, i.e. BCa1F0 is equivalent to BC_1_. All markers that did not show any heterozygous call or were unlinked were ignored and only the highest-ranking linkage groups with more than 100 markers assigned were selected for downstream analysis. Linkage groups from all advanced backcross populations were integrated in JoinMap^®^ to produce one set of seven linkage groups for *Th. bessarabicum*. These were exported and assigned to chromosomes using information from the Axiom^®^ Wheat HD Genotyping Array (Winfield et al. 2012). Erroneous markers that had more than 20% missing data were removed. Each linkage group was divided into various physical segmental blocks based on genotyping and matching GISH data of introgression lines. To obtain the marker order within a segmental block, the marker sequences were used in BLAST against the IWGSC Chinese Spring survey sequence v2 (IWGSC CSS v2; IWGSC 2014) and contigs with the highest or a very high BLAST score on the most likely chromosome, where available for each of the three genomes of wheat, were selected manually from the BLAST output. The genetic positions of the contigs were obtained from the POPSEQ data (Chapman et al. 2015) and used to order the markers in each linkage group (Online Resource 3). The long and short arm of each chromosome was identified and groups were orientated to have the short arm above the long arm. A physical map with chromosome ideograms was produced through MapChart 2.3 (Voorrips 2002). Graphical genotype visualization was performed using Graphical GenoTypes 2.0 (GGT; van Berloo 2008).

### Comparative analysis

Synteny analysis with wheat was carried out using the BLAST positions of the markers on the *Th. bessarabicum* map, obtained as described above. To generate the circos plots, markers on each of the chromosomes on the physical map of *Th. bessarabicum* were separated by an incremental distance of 1 unit. To be able to equate the units of comparison in both species, the marker distance (physical position) on the *Th. bessarabicum* linkage groups and the genetic position of their syntenic contigs in wheat were scaled up by a factor of 1,000,000. Markers corresponding to the same genetic position on a wheat chromosome were grouped at the same physical position within a *Th. bessarabicum* chromosomal segment. Figure 7 was visualized using Circos (v. 0.67; Krzywinski et al. 2009) to observe synteny between *Th. bessarabicum* (physical position in units) and the wheat genome (genetic position in cM).

## Results

### Generation of wheat–*Th*. *bessarabicum* introgressions

A total of 1775 crosses were made between wheat and *Th. bessarabicum* and their derivatives, through both crossing strategies (Fig. 1), leading to the generation of 10,321 crossed seeds (an additional 10,387 self- fertilised seeds were also produced). The number of seeds germinated, plants crossed, cross fertility and seed set are shown in Table 1. Fertility in the backcrosses varied quite substantially among individuals, often ranging from 0% seed set to a high proportion of fertile florets.

**Table 1:** Number of seeds produced and germinated in relation to the number of crosses carried out, across both crossing strategies, for each generation of the introgression programme for *Th. bessarabicum* into wheat

		Seeds sown	Germination rate (%)	Survival to flower (%)	Crosses made	Cross fertility (%)	Crossed seeds produced	Seeds/cross	Self-fertilised seeds produced
Crossing strategy 1	Wheat × *Th. bessarabicum*	–	–	–	184	2.7	5	0.03	0
	F_1_	5	100	100	317	1.6	5	0.02	0
	BC_1_	5	80	60	16	100	127	8	144
	BC_2_	36	81	75	137	93	1748	13	614
	BC_3_	117	89	80	218	97	3389	16	5791
	BC_4_	69	8	23	3	100	32	11	793
	Subtotal	232	–	–	875	–	5306	–	7342
Crossing strategy 2	F_1_	30	93	63	331	24	570	1.7	231
	BC_1_	63	83	65	365	81	2136	6	266
	BC_2_	45	73	73	175	91	1965	11	1261
	BC_3_	356	67	60	29	97	344	12	1287
	Subtotal	494	–	–	900	–	5015	–	3045
	Total	726	–	–	1775	–	10,321	–	10,387

Through crossing strategy 1, five F_1_ hybrid seeds were produced by crossing wheat Paragon *ph1* mutant with *Th. bessarabicum*. All 5 F_1_ seeds were germinated and every year produced was crossed with wheat, i.e. since these amphihaploids were expected to show very little fertility no attempts were made to self-fertilise them. Only 5 BC_1_ seeds were generated, of which only 3 grew to maturity and set seed. Table 1 shows that the F_1_ hybrids had the lowest cross fertility of 1.6% as compared with the backcross generations.

In crossing strategy 2, thirty wheat–*Th*. *bessarabicum* F_1_ synthetic amphidiploids were germinated and of these, 19 plants reached maturity. The cross fertility of these F_1_ plants was much higher at 24%, compared to those from crossing strategy 1. The F_1_ hybrids also produced 231 self-fertilised seeds indicating a moderate level of male and female fertility.

### Development of a FISH-based karyotype for *Th. bessarabicum* chromosomes

**Fig. 2:**
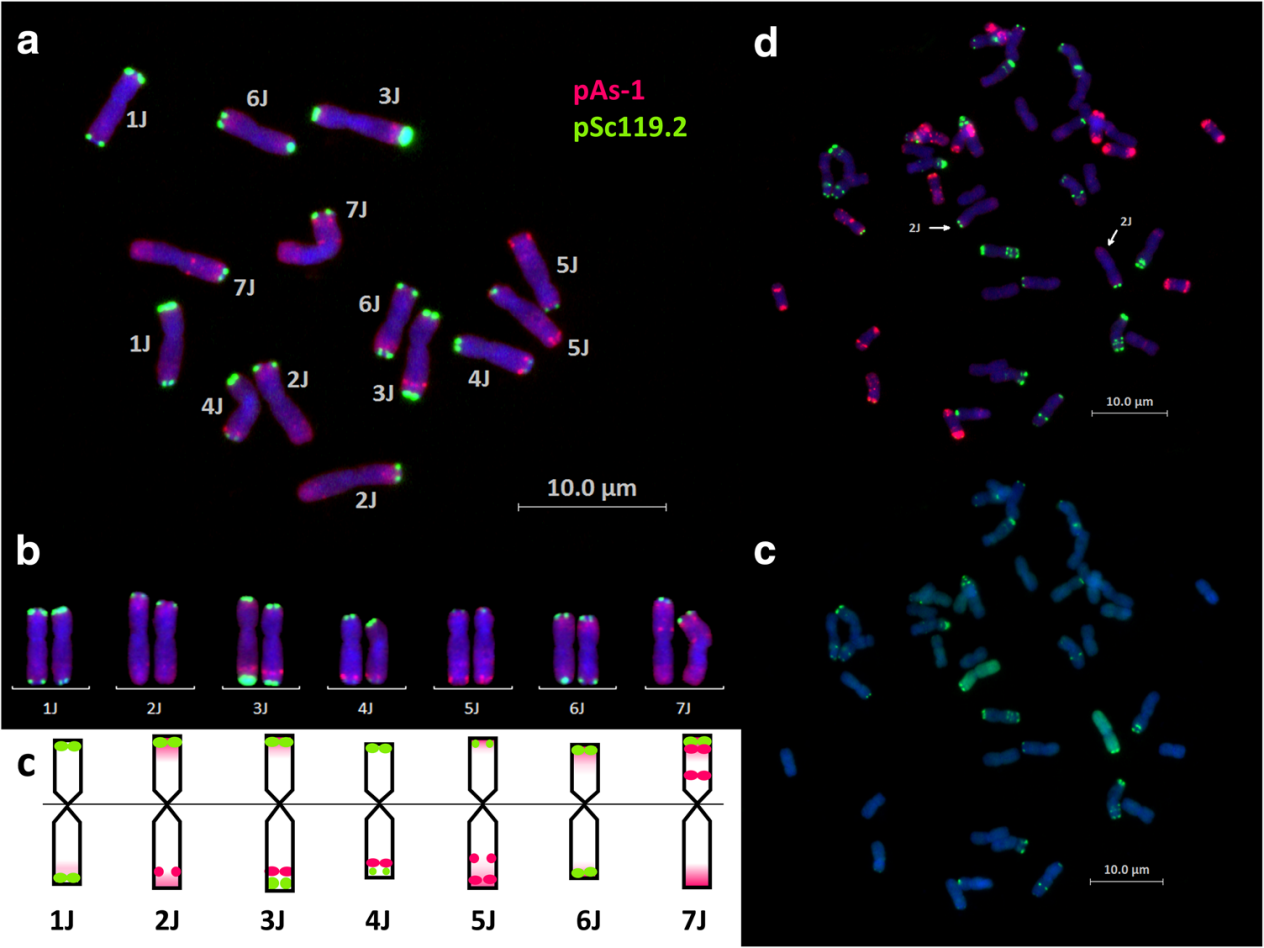
Development of a FISH-based karyotype for *Th. bessarabicum* chromosomes. **a** mc-FISH image of root-tip metaphase spread of chromosomes of *Th. bessarabicum*, accession PI 531712. **b** mc-FISH karyogram of *Th. bessarabicum* chromosomes. **c** Graphical representation of mc-FISH karyogram showing sites of hybridisation on each *Th. bessarabicum* linkage group. **d** mc-FISH image of Line DA2J-1 showing mc-FISH sites on chromosomes 2J indicated by arrows. **e** sequential sc-GISH image of Line DA2J-1 identifying chromosomes 2J in green. mc-FISH shows sites of hybridisation with fluorescence-labelled probes, pSc119.2 (green) and pAs.1 (red). sc-GISH shows hybridisation with fluorescence-labelled *Th. bessarabicum* genomic DNA as probe (green). Chromosomes were stained with DAPI (blue)

To develop a FISH karyotype for *Th. bessarabicum,* mitotic chromosome spreads of *Th. bessarabicum* were analysed by mc-FISH using the two probes Oligop-pAs-1 and Oligo-pSc119.2-1 (Fig. 2a). Results showed that all seven *Th. bessarabicum* chromosomes could be distinguished from each other (Fig. 2b). Allocation of each of the seven pairs of *Th. bessarabicum* chromosomes to Triticeae homoeologous groups was possible based on visual characteristics of mc-FISH patterns. This was done by comparing sequential mc-FISH and sc-GISH of the mitotic spreads of *Th. bessarabicum* disomic addition lines in wheat (Online Resource 1). An example of this comparison is shown for chromosome 2J in DA2J-1 where the mc-FISH banding pattern of 2J (Fig. 2d) could be assigned through identification of chromosomes 2J in the same metaphase spread through sequential sc-GISH analysis (Fig. 2e). After comparison with all disomic addition lines, a mc-FISH karyotype of *Th. bessarabicum* chromosomes was established (Fig. 2c) which will be helpful for the identification of *Th. bessarabicum* chromosomes in a wheat background. As discussed later in the results, the disomic addition line for chromosome 3J (DA3J) was found not to have a whole chromosome 3J and no other source for a 3J disomic addition line was available. Thus, to establish a mc-FISH pattern for 3J, a disomic addition line created through our crossing program was used (Online Resource 1).

mc-FISH showed that both probes, Oligop-pAs-1 and Oligo-pSc119.2-1, mainly hybridized to distal/terminal regions of *Th. bessarabicum* chromosomes (Fig. 2a–c; Online Resource 1). Chromosomes 1J, 3J, 4J, and 6J had Oligo-pSc119.2–1 signals (in green) on terminal regions of both arms, while 2J, 5J, and 7J only had Oligo-pSc119.2-1 signals on terminal regions of the short arms (Fig. 2a–c). Strong Oligo-pAs-1 signals (in red) were observed on the terminal and/or subterminal regions of the long arm of chromosomes 2J, 3J, 4J and 5J and on the subterminal regions of the short arm of chromosome 7J (Fig. 2a–c). In addition, dispersed labelling of Oligo-pAs-1 was also observed on some chromosomes, except for centromeric and most pericentromeric regions. For chromosomes 1J and 6J, this dispersed labelling of Oligo-pAs-1 on the terminal region of the long arm of chromosome 1J and on the terminal region of the short arm of chromosome 6J, is the only distinguishing characteristic apart from the slightly larger size of chromosome 1J (Fig. 2a–c) Therefore, through this mc-FISH hybridization pattern, five chromosome pairs of *Th. bessarabicum* can be confidently identified and two pairs of chromosomes can be reliably identified.

### Cytogenetic analysis of wheat–*Th*. *bessarabicum* introgressions lines

**Fig. 3:**
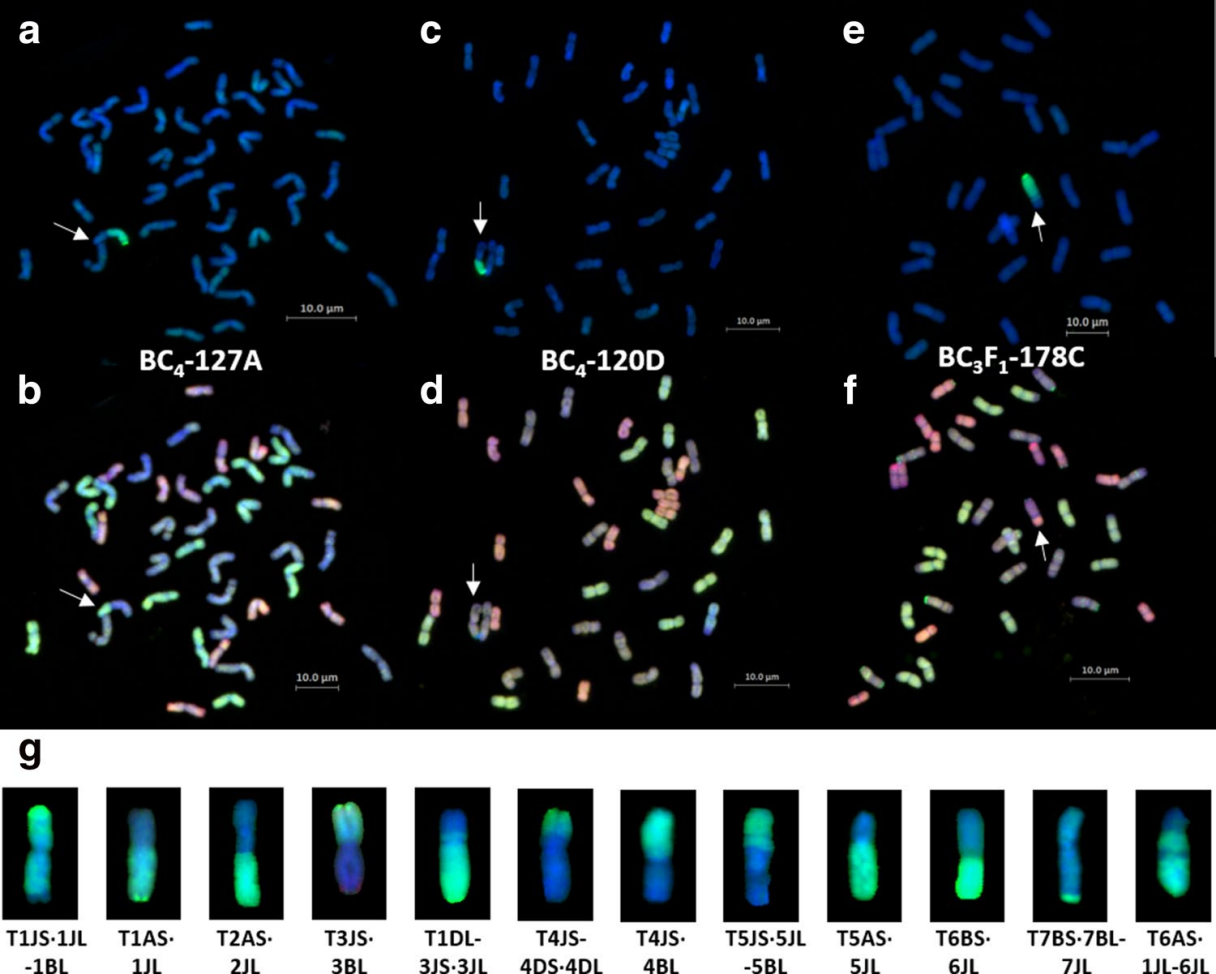
Identification of wheat-*Th. bessarabicum* recombinants through sequential sc-GISH and mc-GISH. Arrows indicate recombinant chromosomes. **a**, **c**, **e** sc-GISH images of lines BC_4_-127A, BC_4_-120D and BC_3_F_1_-178C, respectively, showing *Th. bessarabicum* segments in green. **b**, **d**, **f** mc-GISH images of lines BC_4_-127A, BC_4_-120D and BC_3_F_1_-178C, respectively, showing *Th. bessarabicum* segments (blue) recombined with the A (green), B (blue) and D (red) genomes of wheat, respectively. **g** sc-GISH images of wheat-*Th. bessarabicum* recombinants showing translocation from different J chromosome segments of *Th. bessarabicum* recombined with all three genomes of wheat

To identify wheat–*Th*. *bessarabicum* recombinants in our crossing programme, metaphase spreads of root-tips from 281 lines spread across BC_2_, BC_3_ and BC_4_ generations, from both crossing strategies, were analysed with sequential sc-GISH and mc-GISH as shown in Fig. 3a–f. Most introgression lines were monosomic additions for one or multiple *Th. bessarabicum* chromosomes. Figure 3a, b show sequential GISH images of line BC_4_-127A that showed a Robertsonian translocation from *Th. bessarabicum* recombined with the A genome of wheat. Figure 3c, d show sequential GISH images of line BC_4_-120D that showed a Robertsonian translocation from *Th. bessarabicum* recombined with the B genome of wheat. Figure 3e, f show sequential GISH images of line BC_3_F_1_-178C that showed a large segmental translocation from *Th. bessarabicum* recombined with the D genome of wheat. In total, 12 wheat–*Th*. *bessarabicum* recombinants were identified, which are shown in Fig. 3g. In addition, 13 aberrations were also observed, via GISH, in various J chromosomes in the introgression lines and validated through genotyping with molecular markers (see below). Structural aberrations included deletions (del), telocentrics (tc), chromosome breakages (cb) and translocations between segments of different *Th. bessarabicum* chromosomes (*Tb*–*Tb*). Presence of the *Th. bessarabicum* segment was validated through genotyping as discussed below.

### Molecular marker analysis of wheat–*Th*. *bessarabicum* introgression lines

22,606 SNPs on the Axiom array showed polymorphism between *Th. bessarabicum* and wheat (Table 2). The SNPs appeared to be relatively evenly spread over all seven linkage groups. The Axiom array was used to screen genomic DNA prepared from 422 backcross lines between wheat and *Th. bessarabicum,* from both crossing strategies and 3 wheat–*Th*. *bessarabicum* derivatives from the GRU along with control samples. Genotype calls were generated, and the sample call rate ranged from 87.2 to 99.7% with an average of 98.7% for the 447 samples. The lowest call rates were obtained for the three *Th. bessarabicum* samples with an average of 87.9%. Affymetrix software classified the scores for each of the probes into one of six cluster patterns. However, only those calls classified as PHR were used for genotyping as these are optimum quality.

**Table 2:** Number of SNP markers polymorphic between wheat and *Th. bessarabicum* on the Affymetrix Axiom^®^ Wheat-Relative Genotyping Array for each linkage group of the J genome and final number of SNP markers mapped onto the physical map of the J genome of *Th. bessarabicum* obtained through Poly High Resolution (PHR) calling

	Short arm	Long arm	Both arms	% of total SNP markers	PHR calls on physical map	% of total PHR calls
Linkage group 1	1005	1845	2850	12.6	124	10.8
Linkage group 2	1505	2380	3885	17.2	189	16.4
Linkage group 3	1297	2048	3345	14.8	185	16.1
Linkage group 4	1183	1667	2850	12.6	155	13.5
Linkage group 5	903	2880	3783	16.7	228	19.8
Linkage group 6	1086	1526	2612	11.6	104	9.0
Linkage group 7	1583	1698	3281	14.5	165	14.3
Total	8562	14,044	22,606	100.00	1150	100.0

JoinMap^®^ (van Ooijen 2011) was used to analyse the genotypes of all lines for the PHR SNPs and this led to the establishment of seven linkage groups. In total, the linkage groups were composed of 1150 SNPs and represented the seven chromosomes of *Th. bessarabicum* (Table 2). Linkage group 5 had the highest number of SNPs (19.8%) while linkage group 6 had the lowest (9%).

### Physical mapping of *Th. bessarabicum* chromosomes

**Fig. 4:**
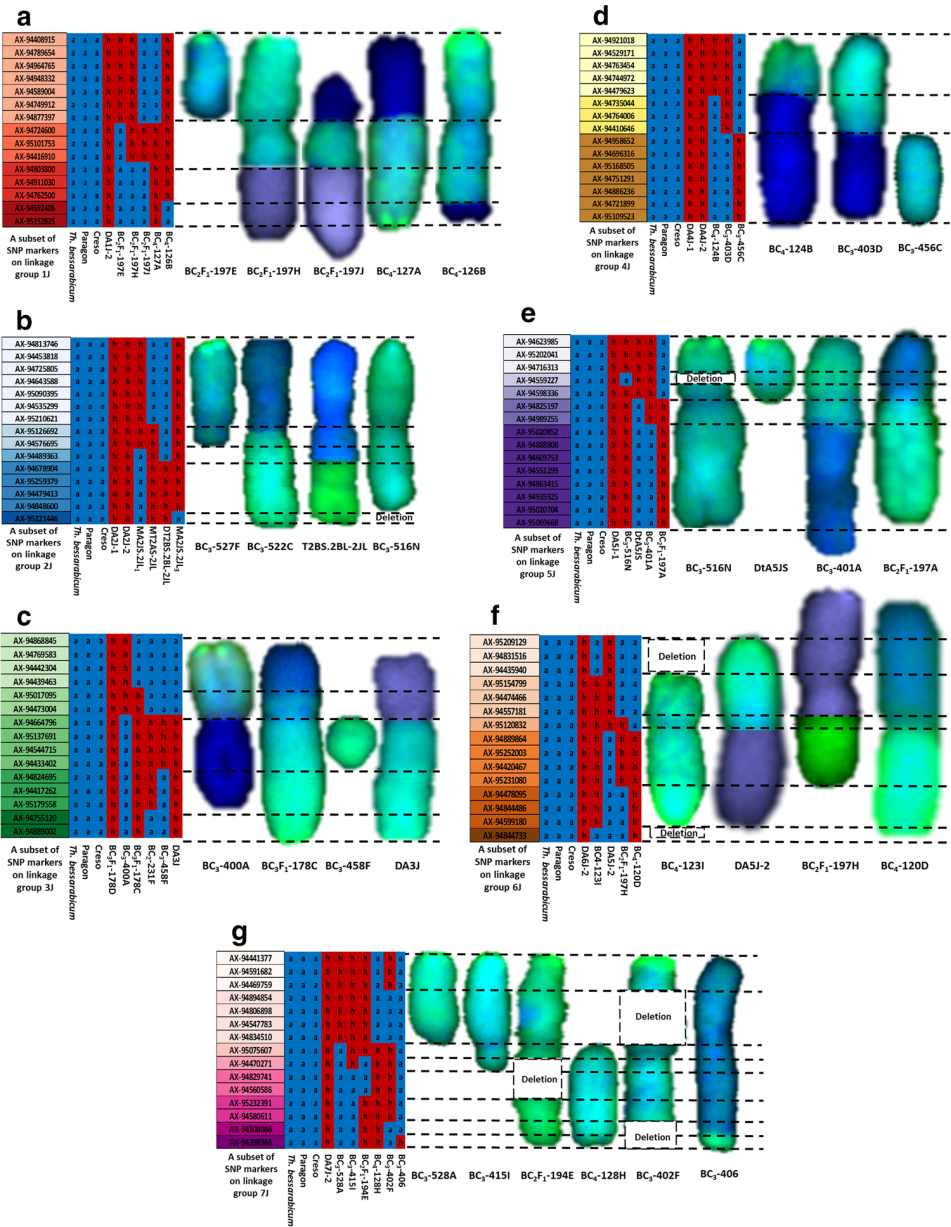
Physical mapping of all seven chromosomes of *Th. bessarabicum* based on combining genotyping results of wheat-*Th. bessarabicum* recombinant lines with sc-GISH images of the recombinant chromosomes and structural aberrations. All chromosome linkage groups show a subset of markers from each segmental block (represented by a different colour) present on that chromosome. Genotyping results show ‘h’ in red representing presence of marker and ‘a’ in blue representing absence of marker. **a** Chromosome 1J is divided into 4 segmental blocks based on the analysis of 3 recombinants and 2 structural aberrations. **b** Chromosome 2J is divided into 5 segmental blocks based on the analysis of 2 recombinants and 2 structural aberrations. **c** Chromosome 3J is divided into 5 segmental blocks based on the analysis of 2 recombinants and 2 structural aberrations. **d** Chromosome 4J is divided into 3 segmental blocks based on the analysis of 2 recombinants and 1 structural aberration. **e** Chromosome 5J is divided into 5 segmental blocks based on the analysis of 2 recombinants and 2 structural aberrations. **f** Chromosome 6J is divided into 6 segmental blocks based on the analysis of 1 recombinant and 3 structural aberrations. **g** Chromosome 7J is divided into 8 segmental blocks based on the analysis of 1 recombinant and 5 structural aberrations

sc-GISH analysis of wheat–*Th*. *bessarabicum* recombinants and structural aberrations was combined with the above genotyping results to establish and/or confirm the order of the markers within each linkage group of *Th. bessarabicum* as shown in Fig. 4a–g. Presence of a heterozygous call for a SNP marker, represented in red and depicted as ‘h’, indicated the presence of *Th. bessarabicum* in a wheat background and a homozygous call represented in blue and depicted as ‘a’, indicated the absence of *Th. bessarabicum* in wheat at that marker. For all linkage groups, genotypes of wheat cvs. Paragon and Creso along with *Th. bessarabicum* were used as negative controls, i.e. to show absence of heterozygous calls. Where possible genotypes of disomic addition lines for the linkage group were used as positive controls, i.e. to show presence of *Th. bessarabicum* via presence of heterozygous calls for the SNP markers of that linkage group. Chromosome segments in green represent *Th. bessarabicum* chromatin which fluoresces due to hybridisation with the *Th. bessarabicum* genomic DNA probe. Chromosome segments in blue represent wheat chromatin and in purple represent *Th. bessarabicum* chromatin from another linkage group. The latter forms part of a structural aberration (*Tb*–*Tb*).

*Linkage group 1* Apart from various monosomic 1J addition lines generated through crossing, three wheat–*Th*. *bessarabicum* recombinant lines BC_2_F_1_-197J, BC_4_-127A and BC_4_-126, and two lines with structural aberrations BC_2_F_1_-197H (*Tb*–*Tb*), BC_2_F_1_-197E (tc) had chromosome 1J specific markers as shown in Fig. 4a. Chromosome 1J had 124 SNP markers, however, a subset of these markers distributed along the chromosome, that detect different introgressed segments of 1J, have been displayed in Fig. 4a. DA1J-2 line was used as the positive control in genotyping.

*Linkage group 2* Chromosome 2J had recombined with wheat only once through our crossing programme resulting in a Robertsonian translocation line. To be able to order the 189 SNP markers on 2J, we used this translocation line, BC_3_-522C, along with two lines with structural aberrations in 2J, BC_3_-527F (cb) and BC_3_-516 N (del), and one wheat–*Th*. *bessarabicum* recombinant (T2BL∙2BS-2JS) obtained from CIMMYT as shown in Fig. 4b. Both DA2J-1 and DA2J-2 were used as positive controls in genotyping.

*Linkage group 3* Chromosome 3J had 185 SNP markers on it and these were ordered through aligning the genotyping data of two wheat–*Th*. *bessarabicum* recombinants, BC_3_-400A and BC_3_F_1_-178C, and three lines with structural aberrations, BC_2_-231F (cb), BC_3_-458F (cb) and DA3J (found to be a *Tb*–*Tb* translocation of 4JS-3JL as described later and shown in Online Resource 4), with their sc-GISH images (could not be obtained for line BC_2_-231F) as shown in Fig. 4c. A disomic addition line for 3J (BC_3_F_1_-178D) generated through our crossing program and confirmed by mc-FISH (Online Resource 1) was used as the positive control for genotyping.

*Linkage group 4* Two wheat–*Th*. *bessarabicum* recombinant lines, BC_4_-124B and BC_3_-403D, along with one line with a structural aberration, BC_3_-456C (tc) were used to order the 155 SNP markers on chromosome 4J as shown in Fig. 4d. DA4J-1 and DA4J-2 were used as the positive controls in genotyping.

*Linkage group 5* Chromosome 5J had the most SNP markers (228) and these were ordered through combining genotyping and sc-GISH analysis of two wheat–*Th*. *bessarabicum* recombinant lines, BC_3_-401A and BC_3_F_1_-197A, and two lines with a structural aberration, BC_3_-516N (del) and DtA5JS (tc), as shown in Fig. 4e. Lines DA5J-1 and DtA5JS were used as positive controls in genotyping where the latter helped in identifying markers on the short arm of chromosome 5J.

*Linkage group 6* 104 SNP markers on chromosome 6J were ordered by aligning the genotyping results and the sc-GISH results from one wheat–*Th*. *bessarabicum* recombinant line showing a Robertsonian translocation, BC_4_-120D, and three lines with structural aberrations, BC_4_-123I (del), BC_3_F_1_-197H (*Tb*–*Tb*) and DA5J-2 (found to be a *Tb*–*Tb* translocation of 6JS-5JL as described later and shown in Online Resource 4), as shown in Fig. 4f. DA6J-2 was used as the positive control in genotyping.

*Linkage group 7* Only one wheat–*Th*. *bessarabicum* recombinant line, showing a very small introgressed segment and detected with one SNP marker, was observed for chromosome 7J which had 165 SNP markers. These markers were ordered through a combined analysis of this recombinant line, BC_3_-406, with five other lines showing structural aberrations, BC_3_-528A (tc), BC_3_-415I (cb), BC_4_-128H (tc), BC_3_F_1_-194E (del) and BC_3_-402F (del), as shown in Fig. 4g. DA7J-2 was used as the positive control in genotyping.

**Fig. 5:**
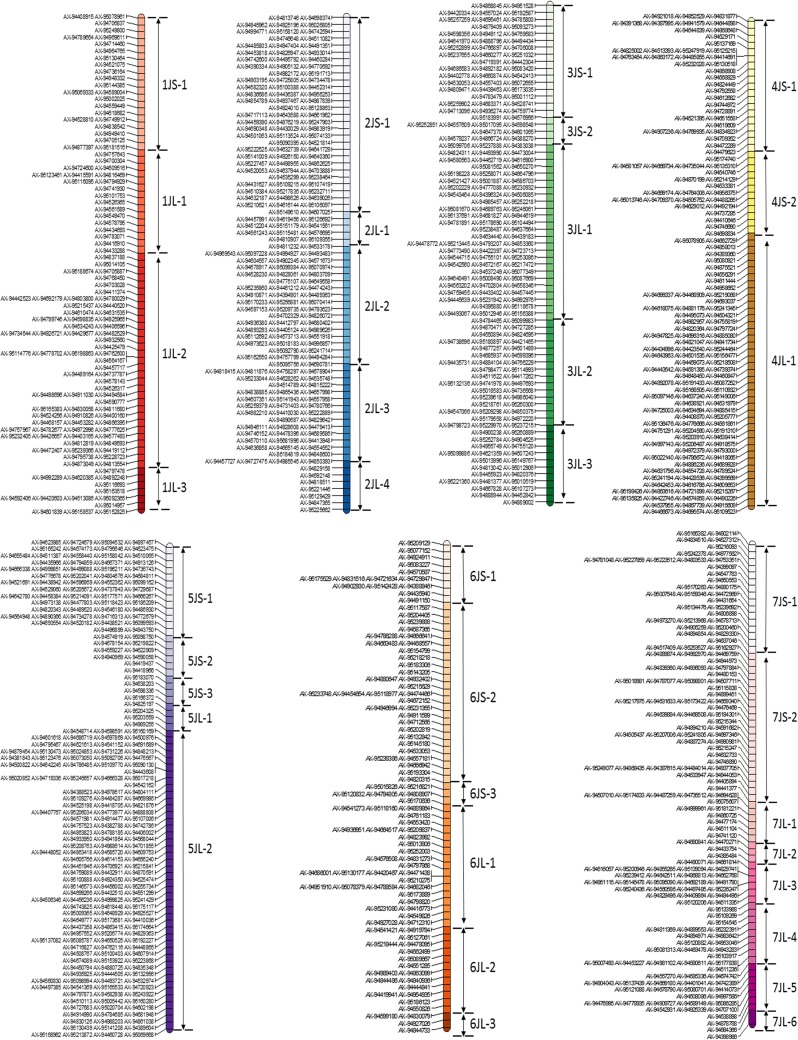
A physical map of *Th. bessarabicum* chromosomes, divided into 36 segmental blocks, containing 1150 SNP markers. The segmental block names are on the right side of the chromosome, with JS in the names representing blocks of markers on the short arm and JL representing blocks of markers on the long arm. Markers within a segmental block were ordered according to wheat POPSEQ data (Chapman et al. 2015). The distribution of markers on each chromosome is also provided in Online Resource 3

Using this methodology, we were able to physically divide the SNP map of *Th. bessarabicum* chromosomes into 36 segmental blocks (Fig. 5). The number and distribution of the SNP markers in each segmental block for chromosomes 1J-7J are shown in Online Resources 2 and 3, respectively. Chromosome 4J was divided into the least number of segmental blocks, i.e. 3, whereas chromosome 7J has the most divisions with 8 segmental blocks. Assuming synteny is conserved to a large extent between wheat and *Th. bessarabicum*, the potential order of the SNP markers within each segmental block was determined through their corresponding POPSEQ genetic position (Chapman et al. 2015). This allowed generation of a putative physical map of *Th. bessarabicum* chromosomes as displayed in Fig. 5 with the short arms of all chromosomes being divided into 14 segmental blocks (JS) and the long arms of all chromosomes being divided into 22 segmental blocks (JL).

**Fig. 6:**
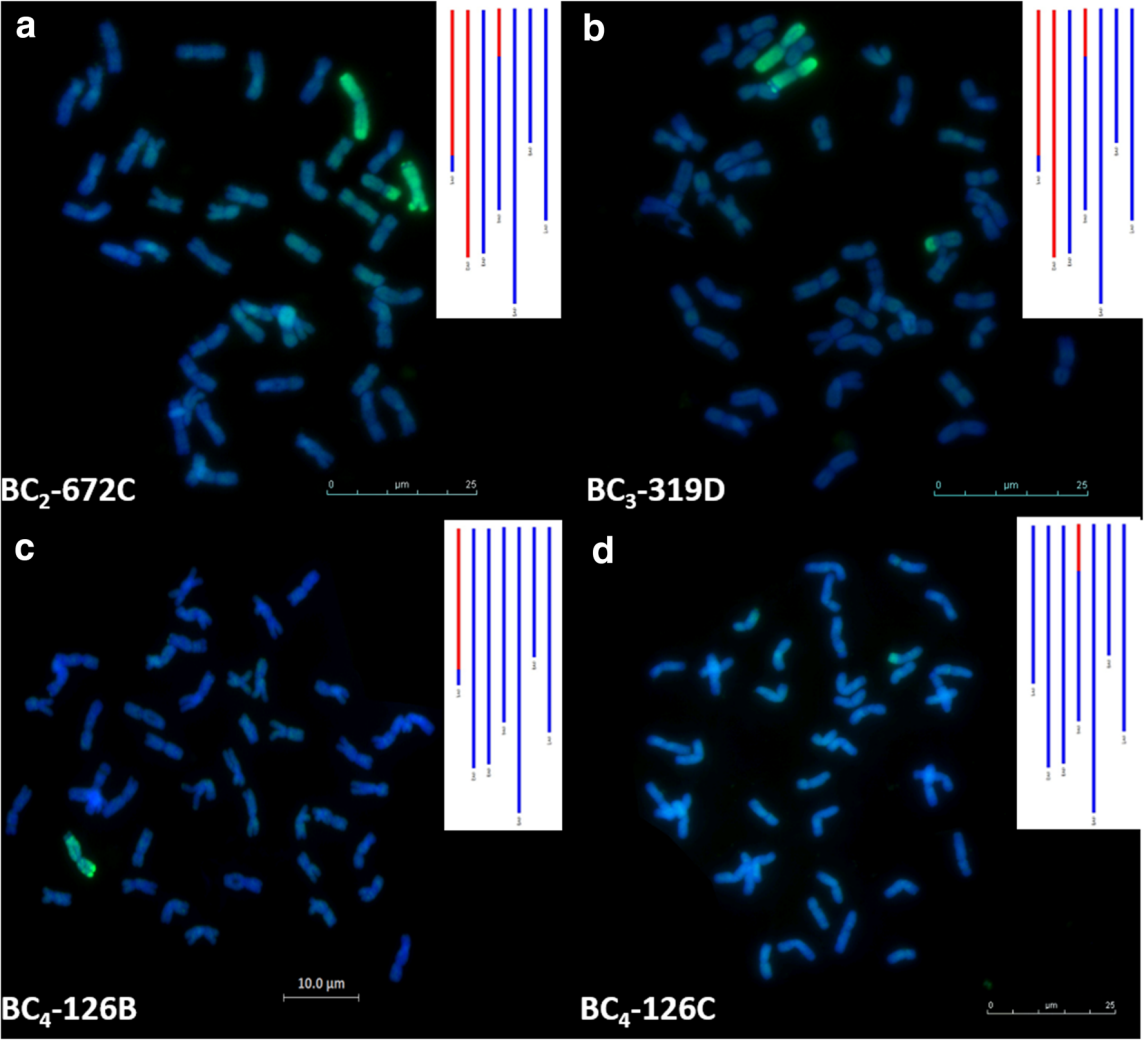
Tracking wheat-*Th. bessarabicum* recombinants within a backcross family through sc-GISH of root-tip metaphase spread of chromosomes and corresponding genotyping data. Red segments in the GGT images represent presence of *Th. bessarabicum* markers and blue segments represent absence of markers. **a** sc-GISH image of line BC_2_-672C showing 3 *Th. bessarabicum* introgressions (green), indicated to be recombinants of chromosomes 1J and 4J and a whole chromosome 2J by the GGT data. **b** sc-GISH image of line BC_3_-319D showing the same 3 introgressions as the BC_2_ parent, confirmed by the GGT data. **c** sc-GISH image of line BC_4_-126B showing one wheat-*Th. bessarabicum* recombinant indicated to be from chromosome 1J by the GGT data. **d** sc-GISH image of line BC_4_-126C showing one wheat-*Th. bessarabicum* recombinant indicated to be from chromosome 4J by the GGT data

A physical SNP map of *Th. bessarabicum* allowed us to track *Th. bessarabicum* introgressions through the backcross generations. Visualisation of the *Th. bessarabicum* segments through sc-GISH validated the genotyping data (indicated through GGT bar diagrams) and the physical map as shown in Fig. 6a–d. A BC_2_ line showed three *Th. bessarabicum* segments in sc-GISH and in its genotyping (indicated in red segments of the GGT diagram in Fig. 6a). Two of these were recombinants with wheat and one was a whole chromosome addition, which were all passed into the BC_3_ plant that was derived from it (Fig. 6b). The wheat–*Th*. *bessarabicum* recombinant chromosomes segregated into different BC_4_ plants as shown in Fig. 6c, d showing recombinants from chromosome 1J and 4J, respectively. The GGT diagrams representing the genotyping results of the BC_3_ and BC_4_ plants match the corresponding sc-GISH images from these lines.

### Comparative analysis of wheat and *Th. bessarabicum* chromosomes

A total of 1150 markers based on the physical map of *Th. bessarabicum* were used in BLAST against the wheat Chinese Spring genome assembly. Contig information for the top hit from each of the three wheat genomes, where available, and for the overall top hit (maximum sequence identity match) was obtained (Online Resource 3). The BLAST results showed that 92.7, 94.4 and 93.5% of the markers had a significant BLAST hit on the A, B and D genomes of wheat, respectively. Of these BLAST hits, 32.5, 37.8 and 32.5% of the markers had an overall top hit on the A, B and D genomes of wheat, respectively, with some showing the same score for the top hit for more than one genome.

**Fig. 7:**
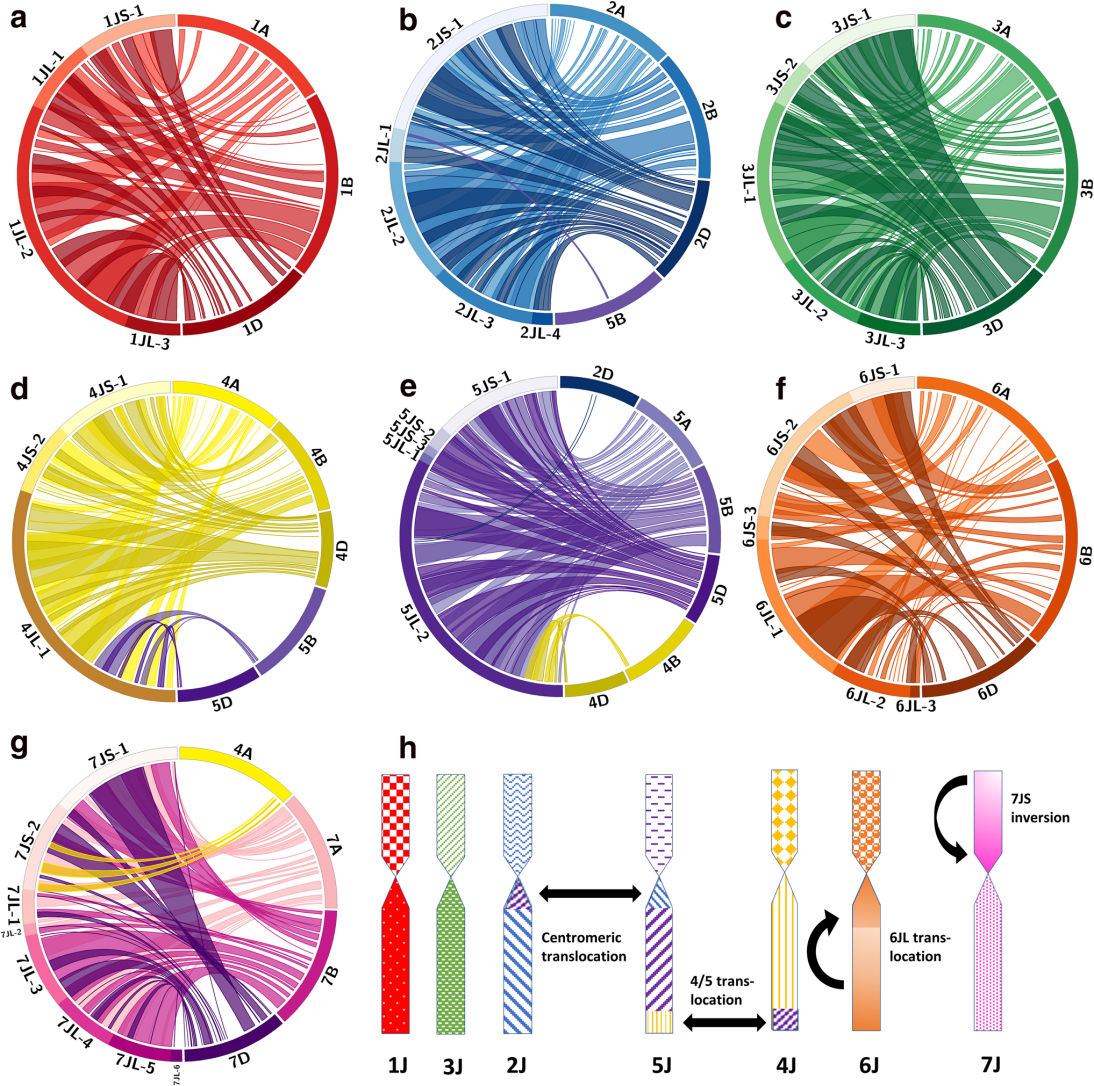
Comparative analysis of synteny between the *Th. bessarabicum* genome and hexaploid wheat. Synteny comparison represented by circos plots with ribbons linking markers between the segmental blocks on the physical map of the J genome and the corresponding genetic positions of those markers on orthologous chromosomes of the A, B and D genomes of hexaploid wheat. **a** Circos plot in hues of red showing synteny between chromosome 1J and group 1 of wheat. **b** Circos plot in hues of blue showing synteny between chromosome 2J and groups 2 and 5 of wheat. **c** Circos plot in hues of green showing synteny between chromosome 3J and group 3 of wheat. **d** Circos plot in hues of yellow showing synteny between chromosome 4J and groups 4 and 5 of wheat. **e** Circos plot in hues of purple showing synteny between chromosome 5J and groups 2,4 and 5 of wheat. **f** Circos plot in hues of orange showing synteny between chromosome 6J and group 6 of wheat. **g** Circos plot in hues of pink showing synteny between chromosome 7J and group 7 of wheat. **h** Graphical representation of the J genome chromosomes and any potential translocations and inversions as discussed in results

Circos plots indicating a significant syntenic relationship between the seven linkage groups of *Th. bessarabicum* and their orthologous chromosomes from each of the three genomes in hexaploid wheat are shown in Fig. 7a–h. Closely spaced links between *Th. bessarabicum* and wheat were grouped together and displayed as ribbons. Chromosomes 1J and 3J show high collinearity between the order of the *Th. bessarabicum* markers and the contigs on the corresponding chromosome groups in wheat on all 3 genomes (Fig. 7a, c, respectively). Chromosome 2J maintained synteny with all three group 2 chromosomes in wheat but also had 2 markers that aligned back to the centromeric region of chromosome 5B in wheat (Fig. 7b) when used in BLAST against the wheat genome assembly. Chromosomes 4J and 5J of *Th. bessarabicum* also maintained synteny with groups 4 and 5 of wheat, respectively (Fig. 7d, e). However, both chromosomes showed an inter-chromosomal translocation on their long arms such as the 4A/5A translocation observed in wheat (Devos et al. 1995; Liu et al. 1992). In addition, chromosome 5J also had 2 markers that aligned to the centromeric region of chromosome 2D in wheat (Fig. 7e) when used in BLAST against the wheat genome assembly. A segment of non-collinear markers that blasted to another region in group 6 of wheat indicated that chromosome 6J showed a translocation within its long arm (Fig. 7f). Chromosome 7J showed an inversion of its short arm as compared to wheat (Fig. 7g) but no translocation with group 4 unlike wheat (Devos et al. 1995; Liu et al. 1992). A summary of all the chromosomal rearrangements in *Th. bessarabicum* is shown in Fig. 7h. This data demonstrates that there is a close syntenic relationship between the A, B and D genomes of wheat and the J genome of *Th. bessarabicum.*

### Genotyping for seedbanks and centric fusions of *Th. bessarabicum* chromosomes

Three wheat–*Th*. *bessarabicum* disomic addition lines WPGS id#28187, WPGS id#28188 and WPGS id#28189 were obtained from GRU with unknown chromosome constitutions. After genotyping on the Axiom^®^ Wheat-Relative Array it was found that WPGS id#28187 was a chromosome 7J disomic addition line and WPGS id#28189 was a chromosome 6J disomic addition line. Genotyping results combined with sequential sc-GISH and mc-FISH showed that WPGS id#28188 was a disomic addition line of a centric fusion of chromosome arms 1JS and 6JL, making it DA1JS·6JL (Online Resource 4).

Genotyping complemented by sc-GISH and mc-FISH analysis also showed that the wheat–*Th*. *bessarabicum* disomic addition lines DA3J and DA5J-2, obtained from CIMMYT, were centric fusions between the arms of chromosomes from different *Th. bessarabicum* groups. DA3J was found to be composed of a disomic addition of a Robertsonian translocation between 4JS and 3JL, whereas DA5J-2 was found to be Robertsonian translocation between 6JS and 5JL (Online Resource 4).

A centric fusion between *Th. bessarabicum* chromosomes was also obtained through our crossing programme where the short arm of chromosome 4J and the long arm of chromosome 7J fused at the centromere in line BC_3_-277C resulting in translocation line T4JS∙7JL as shown in Online Resource 4.

## Discussion

*Th. bessarabicum* is a potentially important source of genetic variation for a range of agronomically important traits (King et al. 1997a; Xu et al. 2009). Many techniques have been used to produce wheat–*Th*. *bessarabicum* translocation lines but here we have exploited the *ph1* mutant approach. Two strategies were used (Fig. 1), one similar to that described by King et al. (2017) where an amphihaploid F_1_ of hexaploid wheat *ph1/ph1* and *Th. bessarabicum* was created and further backcrossed with hexaploid wheat *Ph1/Ph1* (crossing strategy 1) and the second used a durum wheat–*Th*. *bessarabicum* *ph1/ph1* amphidiploid (King et al. 1993a) which was backcrossed with hexaploid wheat *Ph1/Ph1* (crossing strategy 2). Previous studies have highlighted the benefits of the latter approach to obtain wheat–*Th*. *bessarabicum* translocation lines (Mujeeb-Kazi 2006; Mujeeb-Kazi et al. 2013). We modified this approach using a durum wheat instead of hexaploid wheat. This increased the possibility of pairing between the univalent *Th. bessarabicum* chromosomes and the univalent D genome chromosomes from hexaploid wheat used as the female parent in the backcross in the absence of the homologous pairing *Ph1* locus. However, we did not observe any preference for recombination with the D genome in the wheat–*Th*. *bessarabicum* recombinant lines obtained through crossing strategy 2 possibly due to the use of Paragon with *Ph1* locus intact as the first backcross parent in crossing strategy 2. We are currently generating new backcross populations using durum and hexaploid wheat–*Th*. *bessarabicum* *ph1/ph1* amphidiploids and Paragon *ph1/ph1* as the first backcross parent.

Cross fertility in the F_1_ hybrids was 1.6% in crossing strategy 1 which was very low as compared to the cross fertility in the F_1_ hybrids in crossing strategy 2 which was 24% (Table 1). This was expected since the inter-specific F_1_ hybrids generated in crossing strategy 1 were haploid for the A, B, D and J genomes and the frequency of recombination between chromosomes from different genomes is likely to be very low. This would lead to significant infertility in the F_1_, i.e. polyhaploidy would result in the failure of normal disjunction of chromosomes at anaphase I of meiosis leading to low recombination and the production of unviable, unbalanced gametes. Doubling of the chromosome number of sterile wheat–*Th*. *bessarabicum* amphihaploids through colchicine-treatment and their utilisation as the F_1_ plant for further backcrossing in crossing strategy 2 ensured that these synthetic F_1_ amphidiploid lines had much higher fertility than those produced in crossing strategy 1. However, the cross fertility and the average number of seeds set per crossed head in both crossing strategies increased remarkably through the backcross generations as shown in Table 1 indicating restoration of fertility through more stable chromosome numbers in the backcross lines.

The low fertility of the F_1_ hybrids in crossing strategy 1 resulted in the generation of only 5 BC_1_ seeds, of which only 3 grew to maturity and set seed. Hence, if recombination did not occur in later generations, i.e. in the gametes of the BC_1_, BC_2_, BC_3_ and BC_4_ generations, then the total number of introgressions that could be generated was limited to the 3 female F_1_ gametes giving rise to these 3 BC_1_ plants. However, 570 BC_1_ seeds were produced through crossing strategy 2 and even though 41 seeds were backcrossed, many of those BC_1_ seeds have yet to be sown and taken further through the backcrossing program. Due to the low number of BC_1_ plants exploited in this program, the level of interspecific recombination detected through genotyping was also very low such that it was possible to assemble the seven linkage groups of *Th. bessarabicum* (Table 2) but not to produce a genetic map. We identified 12 wheat–*Th*. *bessarabicum* recombinant lines and 13 lines with *Th. bessarabicum* chromosome aberrations through GISH (Figs. 3, 4). These results were combined with genotyping results obtained through the high-throughput Axiom^®^ Wheat-Relative Genotyping Array (Fig. 4) to produce a physical map of *Th. bessarabicum* chromosomes (Fig. 5) consisting of 1150 SNP markers distributed along 36 segmental blocks along the seven J chromosomes of *Th. bessarabicum* (Online Resource 2). Using this physical map, we could further characterise the 25 introgression lines and track them through the backcross generations (Fig. 6). Generating more recombinant lines with different sizes of introgressed segments from *Th. bessarabicum* would facilitate the development of an even more detailed physical map of *Th. bessarabicum.*

Cytogenetic analyses of wheat–*Th*. *bessarabicum* introgression lines have mainly involved sequential sc-GISH and mc-FISH (Patokar et al. 2016; Pu et al. 2015; Qi et al. 2010; Shen et al. 2013) but these studies have used mc-FISH to primarily characterise the wheat chromatin in the introgression lines (Patokar et al. 2016; Qi et al. 2010). Where mc-FISH was used to identify *Th. bessarabicum* chromosomes, it was only reported in studies involving chromosome 4J (Pu et al. 2015; Shen et al. 2013). All *Th. bessarabicum* chromosomes have only been characterised through C-banding until now (Mirzaghaderi et al. 2010). Du et al. (2016) used multiplexing of synthetic FISH oligos to successfully distinguish *Th. bessarabicum* chromosomes from wheat. However, to utilise this kind of FISH technique to identify and characterise wheat-wild relative translocation lines is still quite labour-intensive and requires species-specific sequence information for the wild relative under study which is currently not widely available and is costly to obtain for polyploids. In this study, we used the popular mc-FISH technique with the double probe combination of Oligo-pAs-1 and Oligo-pSc119.2 to generate a FISH karyotype of all seven *Th. bessarabicum* chromosomes. We did this using sequential mc-FISH and sc-GISH on wheat–*Th*. *bessarabicum* disomic addition lines, obtained through seedbanks and validated with SNP markers, where the *Th. bessarabicum* chromosome constitution was known. This was followed by matching the resulting banding patterns to corresponding chromosomes obtained from *Th. bessarabicum* mitotic spreads, thereby assigning a unique mc-FISH pattern to each of the seven pairs of *Th. bessarabicum* chromosomes (Fig. 2; Online Resource 1). It should be noted that the *Th. bessarabicum* accession used in this study was PI 531712, whereas most of the recombinant/translocation lines produced in other studies and the disomic addition lines used for validation in this study were derived from *Th. bessarabicum* accession PI 531711 which could lead to slight variation in the mc-FISH banding patterns. This difference is noticeable in the mc-FISH patterns of chromosome groups 4J and 6J (Online Resource 1) since the Oligo-pAs-1 signals on both these chromosome groups were either very faint or missing as compared to what was expected for accession PI 531712. However, the mc-FISH patterns of 4J and 6J in some of the introgressions lines generated through our crossing programme (shown in insets in Online Resource 1) matched the expected patterns of these chromosome groups since they were generated using the same *Th. bessarabicum* accession (PI 531712) as the one for which the karyotype was being developed.

Comparative analysis between *Th. bessarabicum* and wheat chromosomes showed that macro-synteny between the two species is conserved and the majority of *Th. bessarabicum* chromosome arms are collinear with their orthologous chromosomes in all three genomes in hexaploid wheat. Figure 7d shows markers from the bottom of segmental block 4JL-1 with links to the distal regions of the long arms of chromosomes 5B and 5D and Fig. 7e shows markers from the bottom of segmental block 5JL-2 with links to the distal regions of the long arms of chromosomes 4B and 4D. This indicates a reciprocal translocation between the long arms of chromosomes 4J and 5J which confirms a previous report by King et al. (1994) that indicated that *Th. bessarabicum* has the 4/5 translocation along with *T. urartu* and *Aegilops umbellulata* which had only been previously reported in wheat and rye (Devos et al. 1995; Liu et al. 1992). Since wheat already has the 4A/5A/7B translocation, the links on the A genome of wheat in Fig. 7d, e corresponded to the same chromosome group as *Th. bessarabicum*. This also helped to show that *Th. bessarabicum* did not have the 4/7 translocation as shown in Fig. 7g. Instead chromosome 7J showed an inversion of the small arm as indicated by the twisted links to wheat chromosome 7A, 7B and 7D in Fig. 7g. A break in collinearity with wheat chromosomes 6A, 6B and 6D indicated that chromosome 6JL may have a potential translocation from the distal end of the arm to the proximal end closer to the centromere (Fig. 7f). Presence of markers at the centromeric region of chromosome 2J from the centromeric region of group 5 in wheat and vice versa indicated a potential reciprocal translocation between the centromeric regions of chromosome 2J and 5J as shown in summary Fig. 7h.

Analysis of wheat–*Th*. *bessarabicum* introgression lines have shown several occasions where the *Th. bessarabicum* chromosomes have broken at the centromere producing telocentrics. These telocentrics may recombine with wheat chromosomes to produce Robertsonian translocations, stay univalent as chromosome aberrations or even recombine with other telocentrics from a different chromosome group of *Th. bessarabicum*. We observed that 50% of our 12 wheat–*Th*. *bessarabicum* recombinant lines were Robertsonian translocations (Fig. 3g) with many others reported in the literature (Ardalani et al. 2016; Ghazali et al. 2015; Patokar et al. 2016; Pu et al. 2015). Of the 13 lines identified with chromosome aberrations, 5 had univalent *Th. bessarabicum* telocentrics (Fig. 4). We also observed 4 lines through genotyping and subsequent sequential in situ hybridisation studies where telocentrics from 2 different *Th. bessarabicum* chromosomes had fused at the centromere (Online Resource 4) and such translocations have also been reported in the literature (Chen et al. 2007; Pu et al. 2015; Shen et al. 2013). During meiotic meta-/anaphase I, univalents have a tendency to misdivide (break) across their centromeres. This centric misdivision followed by the fusion of the broken arms from different chromosomes leads to the formation of whole-arm Robertsonian translocations (Robertson 1916) and in wheat, this process has been described in significant detail (Friebe et al. 2005; Lukaszewski 2010; Sears 1952). Thus, potentially due to the monosomic condition of *Th. bessarabicum* chromosomes in most of our backcross lines, we have observed several occurrences of centric fusions between *Th. bessarabicum* and wheat chromosomes and between different *Th. bessarabicum* chromosomes. Due to the preference for small segment introgressions from wild relatives into wheat to avoid linkage drag of negative traits, these Robertsonian translocations can be further backcrossed with wheat to produce small introgressions from *Th. bessarabicum* into wheat (Qi et al. 2010).

We also observed that in the rest of the six wheat–*Th*. *bessarabicum* recombinant lines, that showed homoeologous recombination, 4 were interstitial and 2 were distal recombinants. It has been shown before that most of the recombination occurs in the distal ends of wheat chromosomes (Gill et al. 1993). However, in these limited number of recombinant lines, we observed more frequent recombination between the interstitial regions of wheat and *Th. bessarabicum* chromosomes. This shift of homoeologous recombination towards the centromere has been reported previously for wheat–rye chromosomes (Lukaszewski et al. 2004) and can potentially be a new source for introduction of genetic variation from wild-relatives into wheat.

In this study, we have reported the generation of a new resource of molecular markers that can be used to characterise wheat–*Th*. *bessarabicum* introgression lines and potentially other translocation lines that involve other *Thinopyrum* species containing the J or a closely related genome such as *Thinopyrum intermedium* (J^r^J^r^J^vs^J^vs^StSt) and *Thinopyrum elongatum* (E^e^E^e^) respectively. We have also generated 12 wheat–*Th*. *bessarabicum* recombinant lines that are currently being self-fertilised for further trait analysis. Once stable, homozygous lines have been produced from the recombinant lines, they will be bulked and then made available via the Nottingham/BBSRC Wheat Research Centre website at http://www.nottingham.ac.uk/wisp/index.aspx.

### **Author contribution statement**

SG, CY, SHE, DS, SA, IPK and JK carried out the crossing programme. SG performed the in situ hybridisation experiments. SHE, DS, SA and CY prepared the samples for genotyping and AJB ran the samples on the array. SG analysed the genotyping data, constructed the physical map and performed the comparative genome studies. SG, IPK and JK conceived and designed the experiments. SG wrote the manuscript with assistance from JK and IPK. All authors read and approved the final manuscript.

## Electronic supplementary material

Below is the link to the electronic supplementary material.

**Online Resource 1** mc-FISH and sequential sc-GISH images of disomic addition (DA) lines of *Th. bessarabicum* chromosomes (indicated by arrows) in hexaploid wheat background (PDF 91 kb)

**Online Resource 2** Number of SNP markers in each segmental block (SB) for each linkage group (LG) of the *Th. bessarabicum* physical map of the J genome (PDF 27 kb)

**Online Resource 3** Distribution of SNP markers along each of the seven J chromosomes of *Th. bessarabicum* as depicted on the physical map. Markers within each segmental block were ordered through genetic positions (POPSEQ data; Chapman et al., 2015) of the wheat contigs, for each of the 3 genomes of wheat, obtained through using BLAST against the IWGSC CSS v2 (IWGSC 2014) (XLSX 150 kb)

**Online Resource 4** sc-GISH and sequential mc-FISH images of chromosomes showing Robertsonian translocations (indicated by arrows) between telocentrics from different J chromosomes of *Th. bessarabicum* and validated by genotyping data shown as GGT diagrams in insets (PDF 110 kb)

## References

[CR1] Able JA, Langridge P (2006). Wild sex in the grasses. Trends Plant Sci.

[CR2] Ardalani S, Mirzaghaderi G, Badakhshan H (2016). A Robertsonian translocation from *Thinopyrum bessarabicum* into bread wheat confers high iron and zinc contents. Plant Breeding.

[CR3] Banks PM, Larkin PJ, Bariana HS, Lagudah ES, Appels R, Waterhouse PM, Brettell RIS, Chen X, Xu HJ, Xin ZY, Qian YT, Zhou XM, Cheng ZM, Zhou GH (1995). The use of cell culture for subchromosomal introgressions of barley yellow dwarf virus resistance from *Thinopyrum intermedium* to wheat. Genome.

[CR4] Bie T-D, Cao Y-P, Chen P-D (2007). Mass production of intergeneric chromosomal translocations through pollen irradiation of *Triticum durum*-*Haynaldia villosa* amphiploid. J Integr Plant Biol.

[CR5] Chapman JA, Mascher M, Buluç A, Barry K, Georganas E, Session A, Strnadova V, Jenkins J, Sehgal S, Oliker L, Schmutz J, Yelick KA, Scholz U, Waugh R, Poland JA, Muehlbauer GJ, Stein N, Rokhsar DS (2015). A whole-genome shotgun approach for assembling and anchoring the hexaploid bread wheat genome. Genome Biol.

[CR6] Chen H-F, Qian B-L, Zhuang L-F (2007). Molecular marker analysis on common wheat landrace Chinese spring alien chromosome lines derived from *Thinopyrum bessarabicum* Lӧve. Acta Agron Sin.

[CR7] Chen P, You C, Hu Y, Chen S, Zhou B, Cao A, Wang X (2013). Radiation-induced translocations with reduced *Haynaldia villosa* chromatin at the *Pm21* locus for powdery mildew resistance in wheat. Mol Breed.

[CR8] Danilova TV, Zhang G, Liu W, Friebe B, Gill BS (2017). Homoeologous recombination-based transfer and molecular cytogenetic mapping of a wheat streak mosaic virus and Triticum mosaic virus resistance gene *Wsm3* from *Thinopyrum intermedium* to wheat. Theor Appl Genet.

[CR9] Devos KM, Dubcovsky J, Dvorak J, Chinoy CN, Gale MD (1995). Structural evolution of wheat chromosomes 4A, 5A, and 7B and its impact on recombination. Theor Appl Genet.

[CR10] Du P, Zhuang L, Wang Y, Yuan L, Wang Q, Wang D, Dawadondup Tan L, Shen J, Xu H, Zhao H, Chu C, Qi Z (2016). Development of oligonucleotides and multiplex probes for quick and accurate identification of wheat and *Thinopyrum bessarabicum* chromosomes. Genome.

[CR11] Endo TR (2007). The gametocidal chromosome as a tool for chromosome manipulation in wheat. Chromosome Res.

[CR12] Feuillet C, Langridge P, Waugh R (2008). Cereal breeding takes a walk on the wild side. Trends Genet.

[CR13] Friebe B, Jiang J, Raupp WJ, McIntosh RA, Gill BS (1996). Characterization of wheat-alien translocations conferring resistance to diseases and pests: current status. Euphytica.

[CR14] Friebe B, Zhang P, Linc G, Gill BS (2005). Robertsonian translocations in wheat arise by centric misdivision of univalent at anaphase I and rejoining of broken centromeres during interkinesis of meiosis II. Cytogenet Genome Res.

[CR15] Ghazali S, Mirzaghaderi G, Majdi M (2015). Production of a novel Robertsonian translocation from *Thinopyrum bessarabixum* into bread wheat. Tsitol Genet.

[CR16] Gill KS, Gill BS, Endo TR (1993). A chromosome region-specific mapping strategy reveals gene-rich telomeric ends in wheat. Chromosoma.

[CR17] Gill BS, Friebe BR, White FF (2011). Alien introgressions represent a rich source of genes for crop improvement. Proc Natl Acad Sci USA.

[CR18] Grewal S, Gardiner L-J, Ndreca B, Knight E, Moore G, King IP, King J (2017). Comparative mapping and targeted-capture sequencing of the gametocidal loci in *Aegilops sharonensis*. Plant Genome.

[CR19] Griffiths S, Sharp R, Foote TN, Bertin I, Wanous M, Reader S, Colas I, Moore G (2006). Molecular characterization of *Ph1* as a major chromosome pairing locus in polyploid wheat. Nature.

[CR20] Hassani HS, King IP, Reader SM, Caligari PDS, Miller TE (2010). Can Tritipyrum, a new salt tolerant potential amphiploid, be a successful cereal like Triticale?. J Agr Sci Tech.

[CR21] IWGSC (2014). A chromosome-based draft sequence of the hexaploid bread wheat *Triticum aestivum* genome. Science.

[CR22] Jauhar PP, Chibbar RN (1999). Chromosome-mediated and direct gene transfers in wheat. Genome.

[CR23] King IP, Purdie KA, Orford SE, Reader SM, Miller TE (1993). Detection of homoeologous chiasma formation in *Triticum durum* x *Thinopyrum bessarabicum* hybrids using genomic in situ hybridization. Heredity.

[CR24] King IP, Purdie KA, Rezanoor HN, Koebner RMD, Miller TE, Reader SM, Nicholson P (1993). Characterization of *Thinopyrum bessarabicum* chromosome segments in wheat using random amplified polymorphic DNAs (RAPDs) and genomic in situ hybridization. Theor Appl Genet.

[CR25] King IP, Purdie KA, Liu CJ, Reader SM, Pittaway TS, Orford SE, Miller TE (1994). Detection of interchromosomal translocations within the Triticeae by RFLP analysis. Genome.

[CR26] King IP, Orford SE, Cant KA, Reader SM, Miller TE (1996). An assessment of the salt tolerance of wheat/*Thinopyrum bessarabicum* 5E^b^ addition and substitution lines. Plant Breeding.

[CR27] King IP, Forster BP, Law CC, Cant KA, Orford SE, Gorham J, Reader S, Miller TE (1997). Introgression of salt-tolerance genes from *Thinopyrum bessarabicum* into wheat. New Phytol.

[CR28] King IP, Law CN, Cant KA, Orford SE, Reader SM, Miller TE (1997). Tritipyrum, a potential new salt-tolerant cereal. Plant Breeding.

[CR29] King J, Grewal S, C-y Yang, Hubbart S, Scholefield D, Ashling S, Edwards KJ, Allen AM, Burridge A, Bloor C, Davassi A, da Silva GJ, Chalmers K, King IP (2017). A step change in the transfer of interspecific variation into wheat from *Amblyopyrum muticum*. Plant Biotech J.

[CR30] Krzywinski MI, Schein JE, Birol I, Connors J, Gascoyne R, Horsman D, Jones SJ, Marra MA (2009). Circos: an information aesthetic for comparative genomics. Genome Res.

[CR31] Lapitan NLV, Sears RG, Gill BS (1984). Translocations and other karyotypic structural changes in wheat x rye hybrids regenerated from tissue culture. Theor Appl Genet.

[CR32] Larkin PJ, Scowcroft WR (1981). Somaclonal variation—a novel source of variability from cell cultures for plant improvement. Theor Appl Genet.

[CR33] Liu CJ, Atkinson MD, Chinoy CN, Devos KM, Gale MD (1992). Nonhomoeologous translocations between group 4, 5 and 7 chromosomes within wheat and rye. Theor Appl Genet.

[CR34] Liu W, Danilova TV, Rouse MN, Bowden RL, Friebe B, Gill BS, Pumphrey MO (2013). Development and characterization of a compensating wheat-*Thinopyrum intermedium* Robertsonian translocation with *Sr44* resistance to stem rust (Ug99). Theor Appl Genet.

[CR35] Luan Y, Wang X, Liu W, Li C, Zhang J, Gao A, Wang Y, Yang X, Li L (2010). Production and identification of wheat-*Agropyron cristatum* 6P translocation lines. Planta.

[CR36] Lukaszewski AJ (2010). Behavior of centromeres in univalents and centric misdivision in wheat. Cytogenet Genome Res.

[CR37] Lukaszewski AJ, Rybka K, Korzun V, Malyshev SV, Lapinski B, Whitkus R (2004). Genetic and physical mapping of homoeologous recombination points involving wheat chromosome 2B and rye chromosome 2R. Genome.

[CR38] Masoudi-Nejad A, Nasuda S, McIntosh RA, Endo TR (2002). Transfer of rye chromosome segments to wheat by a gametocidal system. Chromosome Res.

[CR39] McIntyre CL, Pereira S, Moran LB, Appels R (1990). New *Secale cereale* (rye) DNA derivatives for the detection of rye chromosome segments in wheat. Genome.

[CR40] Milne I, Shaw P, Stephen G, Bayer M, Cardle L, Thomas WTB, Flavell AJ, Marshall D (2010). Flapjack—graphical genotype visualization. Bioinformatics.

[CR41] Mirzaghaderi G, Shahsevand Hassani H, Karimzadeh G (2010) C-banded karyotype of *Thinopyrum bessarabicum* and identification of its chromosomes in wheat background. Genet Resour Crop Evol 57

[CR42] Mujeeb-Kazi A (2006) Utilization of genetic resources for bread wheat improvement. In: Genetic resources, chromosome engineering, and crop improvement. CRC Press, pp 61–97

[CR43] Mujeeb-Kazi A, Kazi AG, Dundas I, Rasheed A, Ogbonnaya F, Kishii M, Bonnett D, Wang RR-C, Xu S, Chen P, Mahmood T, Bux H, Farrakh S (2013). Genetic diversity for wheat improvement as a conduit to food security. Adv Agron.

[CR44] Patokar C, Sepsi A, Schwarzacher T, Kishii M, Heslop-Harrison JS (2016). Molecular cytogenetic characterization of novel wheat-*Thinopyrum bessarabicum* recombinant lines carrying intercalary translocations. Chromosoma.

[CR45] Pu J, Wang Q, Shen Y, Zhuang L, Li C, Tan M, Bie T, Chu C, Qi Z (2015). Physical mapping of chromosome 4J of *Thinopyrum bessarabicum* using gamma radiation-induced aberrations. Theor Appl Genet.

[CR46] Qi Z, Du P, Qian B, Zhuang L, Chen H, Chen T, Shen J, Guo J, Feng Y, Pei Z (2010). Characterization of a wheat-*Thinopyrum bessarabicum* (T2JS-2BS·2BL) translocation line. Theor Appl Genet.

[CR47] Rayburn AL, Gill BS (1986). Molecular identification of the D-genome chromosomes of wheat. J Hered.

[CR48] Riley R, Chapman V (1958). Genetic control of the cytologically diploid behaviour of hexaploid wheat. Nature.

[CR49] Robertson WMRB (1916). Chromosome studies. I. Taxonomic relationships shown in the chromosomes of *Tettegidae* and *Acrididiae*: V-shaped chromosomes and their significance in *Acrididae*, *Locustidae* and *Grillidae*: chromosomes and variations. J Morphol.

[CR50] Schneider A, Molnár I, Molnár-Láng M (2008). Utilisation of *Aegilops* (goatgrass) species to widen the genetic diversity of cultivated wheat. Euphytica.

[CR51] Schwarzacher T, Anamthawat-Jónsson K, Harrison GE, Islam AKMR, Jia JZ, King IP, Leitch AR, Miller TE, Reader SM, Rogers WJ, Shi M, Heslop-Harrison JS (1992). Genomic in situ hybridization to identify alien chromosomes and chromosome segments in wheat. Theor Appl Genet.

[CR52] Sears ER (1952). Misdivision of univalents in common wheat. Chromosoma.

[CR53] Shen Y, Shen J, Dawadondup Zhuang L, Wang Y, Pu J, Feng Y, Chu C, Wang X, Qi Z (2013). Physical localization of a novel blue-grained gene derived from *Thinopyrum bessarabicum*. Mol Breed.

[CR54] Tiwari VK, Wang S, Sehgal S, Vrána J, Friebe B, Kubaláková M, Chhuneja P, Doležel J, Akhunov E, Kalia B, Sabir J, Gill BS (2014). SNP discovery for mapping alien introgressions in wheat. BMC Genomics.

[CR55] Tiwari VK, Wang S, Danilova T, Koo DH, Vrana J, Kubalakova M, Hribova E, Rawat N, Kalia B, Singh N, Friebe B, Dolezel J, Akhunov E, Poland J, Sabir JS, Gill BS (2015). Exploring the tertiary gene pool of bread wheat: sequence assembly and analysis of chromosome 5M(g) of *Aegilops geniculata*. Plant J.

[CR56] van Berloo R (2008). GGT 2.0: versatile software for visualization and analysis of genetic data. J Hered.

[CR57] Van Ooijen JW (2011). Multipoint maximum likelihood mapping in a full-sib family of an outbreeding species. Genet Res.

[CR58] Voorrips RE (2002). MapChart: software for the graphical presentation of linkage maps and QTLs. J Hered.

[CR59] Wilkinson PA, Winfield MO, Barker GLA, Allen AM, Burridge A, Coghill JA, Edwards KJ (2012). CerealsDB 2.0: an integrated resource for plant breeders and scientists. BMC Bioinform.

[CR60] Wilkinson PA, Winfield MO, Barker GLA, Tyrrell S, Bian X, Allen AM, Burridge A, Coghill JA, Waterfall C, Caccamo M, Davey RP, Edwards KJ (2016). CerealsDB 3.0: expansion of resources and data integration. BMC Bioinform.

[CR61] William MDHM, Mujeeb-Kazi A (1993). *Thinopyrum bessarabicum*: biochemical and cytological markers for the detection of genetic introgression in its hybrid derivatives with *Triticum aestivum* L. Theor Appl Genet.

[CR62] William MDHM, Mujeeb-Kazi A (1995). Biochemical and molecular diagnostics of *Thinopyrum bessarabicum* chromosomes in *Triticum aestivum* germplasm. Theor Appl Genet.

[CR63] Winfield MO, Wilkinson PA, Allen AM, Barker GLA, Coghill JA, Burridge A, Hall A, Brenchley RC, D’Amore R, Hall N, Bevan MW, Richmond T, Gerhardt D, Jeffrey A, Jeddeloh JA, Edwards KJ (2012). Targeted re-sequencing of the allohexaploid wheat exome. Plant Biotech J.

[CR64] Winfield MO, Allen AM, Burridge AJ, Barker GLA, Benbow HR, Wilkinson PA, Coghill J, Waterfall C, Davassi A, Scopes G, Pirani A, Webster T, Brew F, Bloor C, King J, West C, Griffiths S, King I, Bentley AR (2016). High-density SNP genotyping array for hexaploid wheat and its secondary and tertiary gene pool. Plant Biotech J.

[CR65] Xu SS, Jin Y, Klindworth DL, Wang RR-C, Cai X (2009). Evaluation and characterization of seedling resistances to stem rust Ug99 races in wheat–alien species derivatives. Crop Sci.

[CR66] Zhang JY, Li XM, Wang RRC, Cortes A, Rosas V, Mujeeb-Kazi A (2002). Molecular cytogenetic characterization of Eb-genome chromosomes in *Thinopyrum bessarabicum* disomic addition lines of bread wheat. Int J Plant Sci.

[CR67] Zhang H, Bian Y, Gou X, Zhu B, Xu C, Qi B, Li N, Rustgi S, Zhou H, Han F, Jiang J, von Wettstein D, Liu B (2013). Persistent whole-chromosome aneuploidy is generally associated with nascent allohexaploid wheat. Proc Natl Acad Sci USA.

[CR68] Zhang H, Mittal N, Leamy LJ, Barazani O, Song B-H (2017). Back into the wild—apply untapped genetic diversity of wild relatives for crop improvement. Evol Appl.

[CR69] Zhao R, Wang H, Xiao J, Bie T, Cheng S, Jia Q, Yuan C, Zhang R, Cao A, Chen P, Wang X (2013). Induction of 4VS chromosome recombinants using the CS *ph1b* mutant and mapping of the wheat yellow mosaic virus resistance gene from *Haynaldia villosa*. Theor Appl Genet.

[CR70] Zhuang L, Qi Z, Chen P, Feng Y, Liu D (2004). Development and identification of *Triticum aestivum* L.–*Thinopyrum bessarabicum* Love chromosome translocations. Agric Sci China.

