# Supplementary material for: Characterisation of Thinopyrum bessarabicum chromosomes through genome-wide introgressions into wheat
Source: Theor Appl Genet. 2017 Nov 3;131(2):389–406. doi: 10.1007/s00122-017-3009-y (PMC5787220; doi:10.1007/s00122-017-3009-y)
Supplement: Supplementary file 1 — Online Resource 1 mc-FISH and sequential sc-GISH images of disomic addition (DA) lines of Th. bessarabicum chromosomes (indicated by arrows) in hexaploid wheat background (PDF 91 kb) [file 122_2017_3009_MOESM1_ESM.pdf]

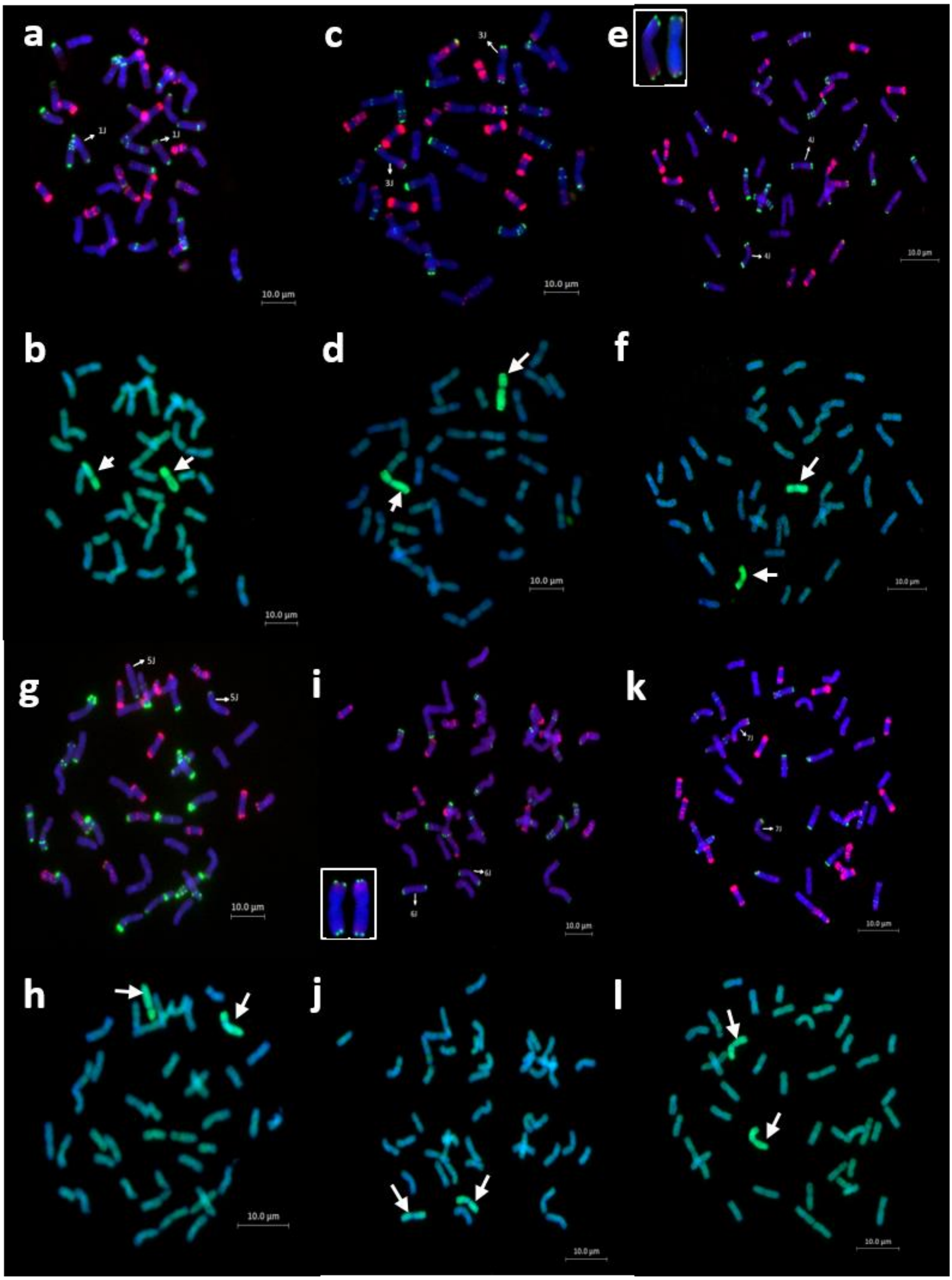

Online Resource 1 mc-FISH and sequential sc-GISH images of disomic addition (DA) lines of *Th. bessarabicum* chromosomes (indicated by arrows) in hexaploid wheat background. **a, b** DA1J-2. **c, d** BC_3F_1-178D (used in rplace of DA3J). **e, f** DA4J-2 with inset showing mc-FISH pattern of chromosomes 4J from other back-cross lines produced through our crossing programme. **g, h** DA5J-1. **i, j** DA6J-2 with inset showing mc-FISH pattern of chromosomes 6J from other back-cross lines produced through our crossing programme. **k, l** DA7J-2. mc-FISH shows sites of hybridisation with fluorescence-labelled probes, pSc119.2 (green) and pAs.1 (red). sc-GISH shows hybridisation with fluorescence-labelled *Th. bessarabicum* genomic DNA as probe (green). Chromosomes were stained with DAPI (blue)
