# Supplementary material for: Characterisation of Thinopyrum bessarabicum chromosomes through genome-wide introgressions into wheat
Source: Theor Appl Genet. 2017 Nov 3;131(2):389–406. doi: 10.1007/s00122-017-3009-y (PMC5787220; doi:10.1007/s00122-017-3009-y)
Supplement: Supplementary file 2 — Online Resource 2 Number of SNP markers in each segmental block (SB) for each linkage group (LG) of the Th. bessarabicum physical map of the J genome (PDF 27 kb) [file 122_2017_3009_MOESM2_ESM.pdf]

Online Resource 2 Number of SNP markers in each segmental block (SB) for each linkage group (LG) of the *Th. bessarabicum* physical map of the J genome

	**SB 1**	**SB 2**	**SB 3**	**SB 4**	**SB 5**	**SB 6**	**SB 7**	**SB 8**	**Total**
**LG 1J**	25	19	67	13	-	-	-	-	124
**LG 2J**	80	13	48	41	7	-	-	-	189
**LG 3J**	46	18	63	35	23	-	-	-	185
**LG 4J**	38	23	94	-	-	-	-	-	155
**LG 5J**	59	10	4	4	151	-	-	-	228
**LG 6J**	14	33	5	33	17	2	-	-	104
**LG 7J**	46	32	12	4	26	22	22	1	165
