# Supplementary material for: Characterisation of Thinopyrum bessarabicum chromosomes through genome-wide introgressions into wheat
Source: Theor Appl Genet. 2017 Nov 3;131(2):389–406. doi: 10.1007/s00122-017-3009-y (PMC5787220; doi:10.1007/s00122-017-3009-y)
Supplement: Supplementary file 4 — Online Resource 4 sc-GISH and sequential mc-FISH images of chromosomes showing Robertsonian translocations (indicated by arrows) between telocentrics from different J chromosomes of Th. bessarabicum and validated by genotyping data shown as GGT diagrams in insets (PDF 110 kb) [file 122_2017_3009_MOESM4_ESM.pdf]

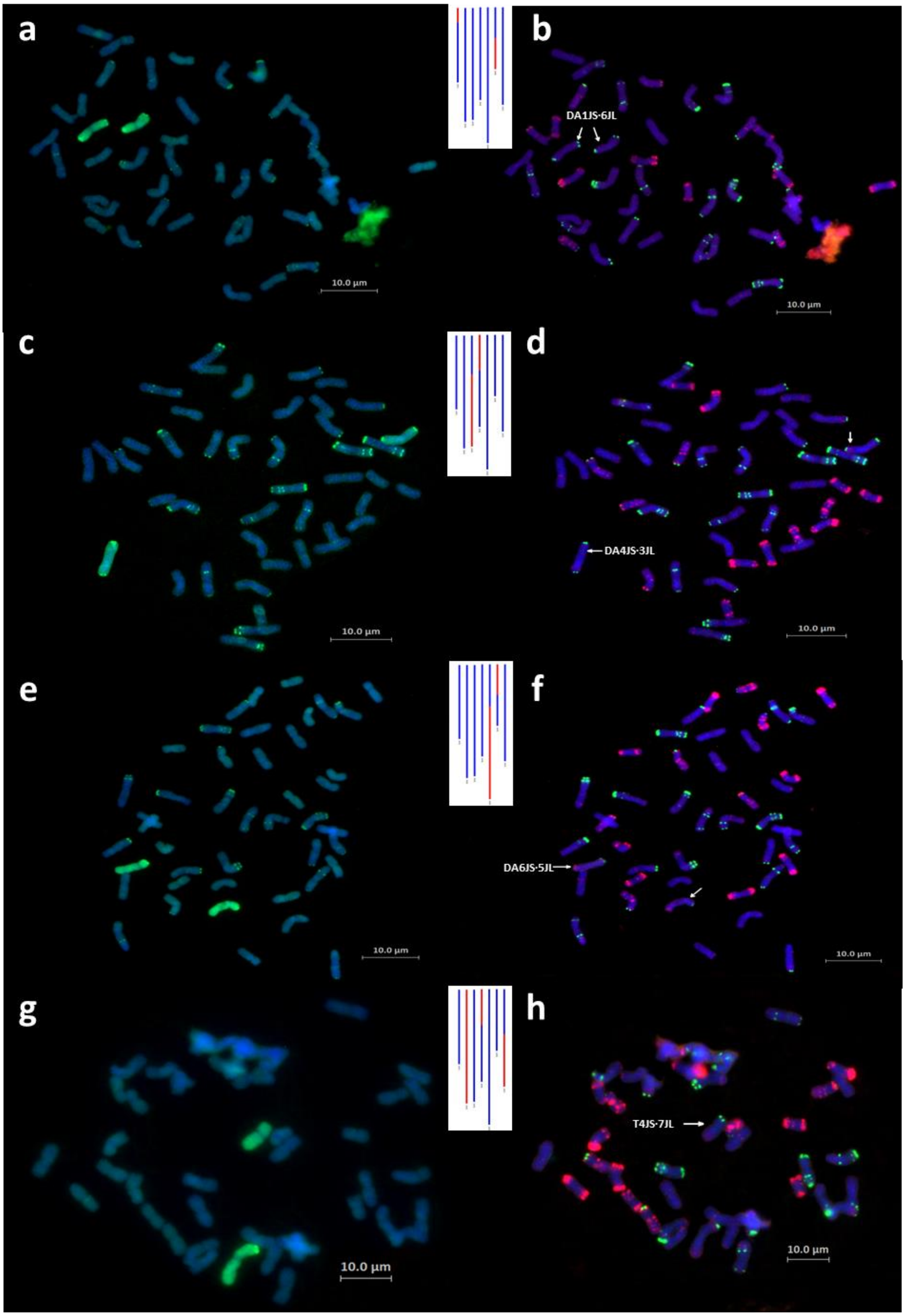

Online Resource 4 sc-GISH and sequential mc-FISH images of chromosomes showing Robertsonian translocations (indicated by arrows) between telocentrics from different J chromosomes of *Th. bessarabicum* and validated by genotyping data shown as GGT diagrams in insets. **a, b** WPGS id#28188 was found to be DA1JS·6JL. **c, d** DA3J was found to be DA4JS·3JL. **e, f** DA5J-2 was found to be DA6JS·5JL. **g, h** BC_3-277C was found to have translocation T4JS·7JL.
